# Anosmin-1 activates vascular endothelial growth factor receptor and its related signaling pathway for olfactory bulb angiogenesis

**DOI:** 10.1038/s41598-019-57040-3

**Published:** 2020-01-13

**Authors:** Shoko Matsushima, Akio Shimizu, Manami Kondo, Hirotsugu Asano, Nobuhiro Ueno, Hironao Nakayama, Naoko Sato, Masahiro Komeno, Hisakazu Ogita, Misuzu Kurokawa-Seo

**Affiliations:** 1 Division of Life Sciences, Department of Molecular Biosciences, Kyoto Sangyo University, Kyoto, 603-8555 Japan; 2 Division of Molecular Medical Biochemistry, Department of Biochemistry and Molecular Biology, Shiga University of Medical Science, Otsu, Shiga, 520-2192 Japan; 3 Department of Medical Science and Technology, Hiroshima International University, Higashi-Hiroshima, Hiroshima, 739-2695 Japan; 4 Department of Pediatrics, Graduate School of Medicine, University of Tokyo, Tokyo, 113-8655 Japan

**Keywords:** Body patterning, Endocrine reproductive disorders

## Abstract

Anosmin-1 is a secreted glycoprotein encoded by the *ANOS1* gene, and its loss of function causes Kallmann syndrome (KS), which is characterized by anosmia and hypogonadism due to olfactory bulb (OB) dysfunction. However, the physiological function of anosmin-1 remains to be elucidated. In KS, disordered angiogenesis is observed in OB, resulting in its hypoplasia. In this study, we examined the involvement of anosmin-1 in angiogenic processes. Anosmin-1 was detected on the vessel-like structure in OB of chick embryos, and promoted the outgrowth of vascular sprouts as shown by assays of OB tissue culture. Cell migration, proliferation, and tube formation of endothelial cells were induced by treatment with anosmin-1 as well as vascular endothelial growth factor-A (VEGF-A), and further enhanced by treatment with both of them. We newly identified that anosmin-1 activated VEGF receptor-2 (VEGFR2) by binding directly to it, and its downstream signaling molecules, phospholipase Cγ1 (PLCγ1) and protein kinase C (PKC). These results suggest that anosmin-1 plays a key role in the angiogenesis of developing OB through the VEGFR2–PLCγ1–PKC axis by enhancing the VEGF function.

## Introduction

Anosmin-1 is a 100-kDa secreted glycoprotein and is an axon guidance molecule^1–3^. The protein is expressed in the brain, olfactory bulb (OB), retina, spinal cord, and kidney in the developmental stage^3–7^. Orthologs of *ANOS1*, which encodes anosmin-1 and is located on the short arm of the X chromosome^8,9^, have been identified in both vertebrates (e.g., human, chicken, and zebrafish) and invertebrates (e.g., *Drosophila* and *Caenorhabditis elegans*)^10–14^, but not in rodents. This is most likely due to the highly divergent nucleotide sequence. This situation has prevented the generation of a mouse model with knockout of this gene.

The *ANOS1* gene is responsible for the X-linked form of Kallmann syndrome (KS), as revealed by the identification of mutations in this gene in affected families^15^. Most of these are deletion, frameshift, or nonsense mutations, and are expected to affect the overall length or composition of the gene product. In principle, this is sufficient to account for the disease symptoms^6^. Anosmia is observed in KS^16^, and is a consequence of the partial or complete lack of OB development. KS also causes hypogonadism because of deficiency in the gonadotropin-releasing hormone (GnRH), which presumably results from the failure of the migration of neuroendocrine GnRH cells from the olfactory epithelium to the forebrain during development^6^. The hypoplasia of OBs and deficiency of GnRH could be induced by disordered olfactory nerve axonal elongation, which is considered to be facilitated by anosmin-1. However, the underlying mechanism by which anosmin-1 contributes to OB development is still not well understood.

Vascular and neuronal cells interact with each other in different ways in the brain. Constant adjustments of the vasculature are required to match the perfusion demands imposed by dynamic changes in neuronal activities. The vasculature may also provide essential neurotrophic factors and participate in the generation of an appropriate niche for neurogenesis. The vascular endothelial growth factor (VEGF)-induced activation of VEGF receptor (VEGFR) is a process that is critical for the generation of mature vasculature in embryos as well as in adults^17^. Blood vessels are reported to play an important role in maintaining OB because the radially migrating cells that constitute OB use blood vessels as a scaffold for their migration^18^. Although the VEGF–VEGFR axis plays an important role in vasculogenesis and angiogenesis, it might not be sufficient to generate mature vasculature for the appropriate development of tissues and organs^19–21^. Based on these findings, we hypothesized that, besides VEGF, anosmin-1 may associate with VEGF receptor for the enhancement of angiogenesis to develop OB. Moreover, we proposed that the loss of anosmin-1 in KS causes the hypoplasia of OB via disordered angiogenesis.

In this study, we found that anosmin-1 promoted migration, proliferation, and tube formation of vascular endothelial cells, resulting in increased angiogenesis in OB. We proved for the first time that anosmin-1 directly interacted with and activated VEGFR2, and induced the activation of the phospholipase Cγ1 (PLCγ1)–protein kinase C (PKC) signaling downstream of VEGFR2 to facilitate the angiogenic process.

## Results

### Anosmin-1 localizes on the endothelial cells of the vessels in OB and it induces angiogenesis

**Figure 1:**
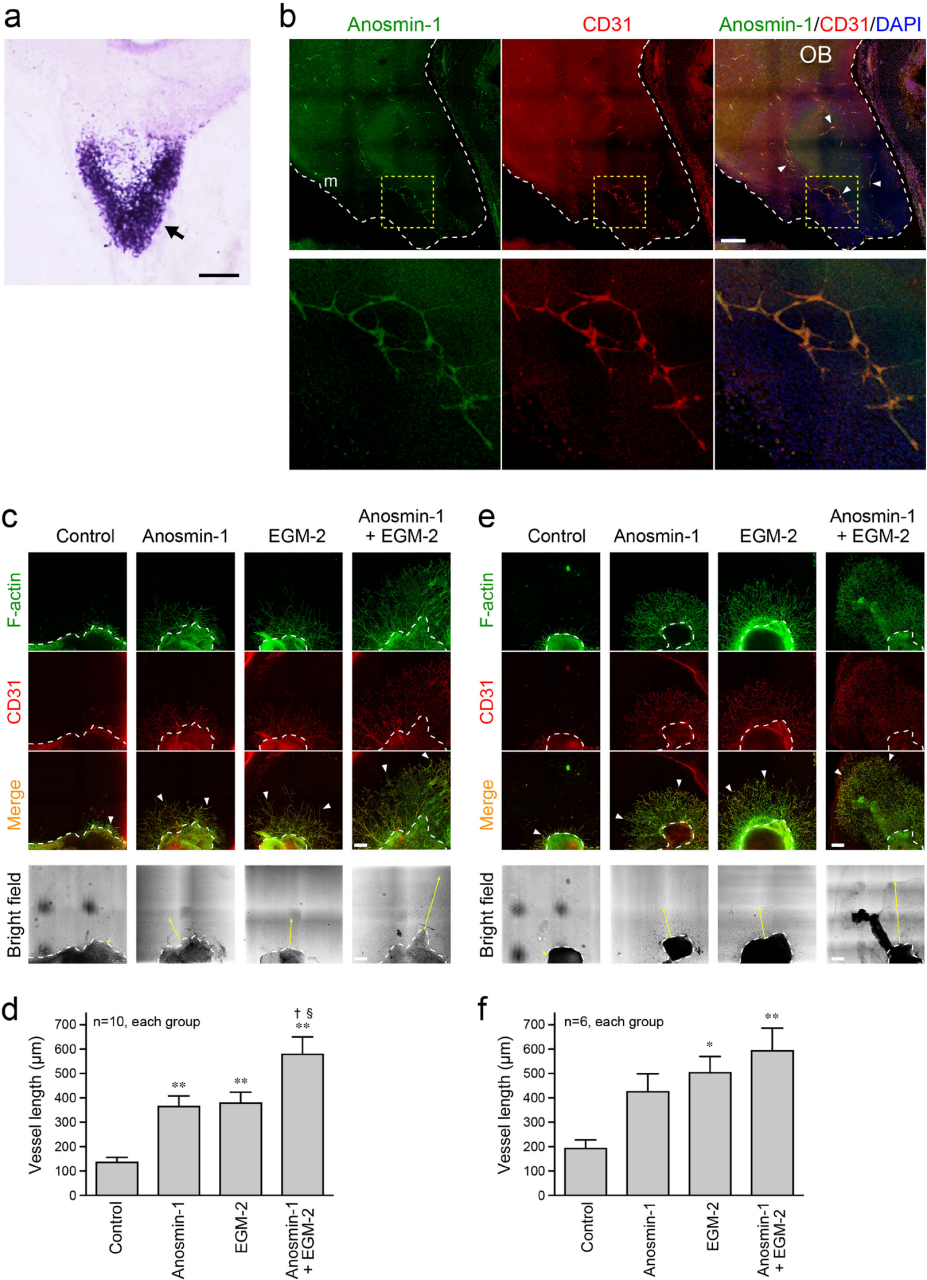
Localization of anosmin-1 in chick embryonic OB and angiogenic activity of anosmin-1 in tissue culture. (**a**) The expression of anosmin-1 mRNA in chick embryonic OB. Coronal OB section of the chick embryo at E10 was hybridized with the *ANOS1* antisense RNA probe. The mitral cell layer was strongly labeled (arrow). Scale bar: 200 μm. (**b**) Immunohistochemical analysis of OB in the chick embryo at E10. Frozen sections of OBs were stained with the anti-anosmin-1 (green) and anti-CD31 (red) Abs. White dotted lines indicate the inner area of OB, and yellow dotted squares are the regions that are magnified and shown below. m: the mitral cell layer of OB. Arrowheads: colocalization of the signals for anosmin-1 and CD31. Scale bar: 200 μm. (**c–f**) Tissue culture of OB (**c**) and pulmonary artery (**e**) from the chick embryo at E11 and E12, respectively. The OB and pulmonary artery specimens in the culture media were treated with anosmin-1 and/or EGM-2 or without supplements (Control) for 3 days, followed by staining with phalloidin for F-actin (green) and with the anti-CD31 Ab for endothelial cells (red). Arrowheads indicate the sprouting vessel-like structures, and dotted lines indicate OB (**c**) and pulmonary artery (**e**). Yellow arrows in the bright field indicate the stretch of vessel-like structures. Scale bars: 250 μm. Graphs indicate the lengths of sprouting vessel-like structures from OB (**d**) or pulmonary artery (**f**). *P < 0.05 and **P < 0.01 vs. Control; ^†^P < 0.05 vs. anosmin-1; ^§^P < 0.05 vs. EGM-2.

At the beginning of our study, we confirmed the expression of anosmin-1 in OB by *in situ* hybridization using chick embryos on embryonic day (E) 10, and found that anosmin-1 was highly expressed in the mitral cell layer and, to a lesser extent, in the inner area of OB (Fig. 1a). To examine the localization of anosmin-1 in OB in detail, we performed an immunohistochemical analysis. In the OB of the E10 chick embryos, the signal for anosmin-1 was present on the CD31-positive cells (Fig. 1b, arrowheads). CD31 is a marker of vascular endothelial cells. Three-dimensional images were generated by the pictures captured in a Z-stack mode (Supplementary Movie S1). The specificity of the anti-anosmin-1 antibody (Ab) that we used has already been certified^22^, and was further tested by the following methods: 1) recombinant chick anosmin-1 protein was added during the incubation of samples with the anti-anosmin-1 Ab (quenching); 2) samples were incubated without the anti-anosmin-1 Ab; and 3) the mouse OB, which does not express anosmin-1 due to loss of the *anosmin-1* gene in the mouse, was incubated with the anti-anosmin-1 Ab. In all experiments, the anosmin-1 signal was not visualized (Supplementary Fig. S1), which confirms that the anti-anosmin-1 Ab specifically detects the protein in the immunohistochemical analysis. These results suggest that anosmin-1 localizes on the vasculature in OB.

To examine the involvement of anosmin-1 in angiogenesis, we performed a tissue culture assay using OBs and the pulmonary arteries of E11 and E12 chick embryos. The OB specimen isolated from the E11 embryo was treated with anosmin-1 and/or endothelial cell basal medium (EBM)-2 containing optimal growth factors and supplements (EGM-2). The outgrowth of vascular sprouts, which were positive for CD31 staining, was remarkably induced in the presence of anosmin-1 or EGM-2, compared with that in the control (Fig. 1c,d). Treatment with both anosmin-1 and EGM-2 further extended the vascular sprouts. Similar results were observed in the pulmonary artery samples isolated from the E12 embryo (Fig. 1e,f). These results suggest that anosmin-1 exerts the angiogenic activity in different types of tissues including OB and has additive effects on the activity in the EGM-2 culture media. Furthermore, we examined the role of endogenous anosmin-1 secreted from OB in the OB angiogenesis by knockdown of anosmin-1. When the OB specimen was treated with the anosmin-1 siRNA, the expression of anosmin-1 was inhibited (Supplementary Fig. S2a), and the outgrowth of vascular sprouts was significantly reduced, compared with that treated with the control siRNA (Supplementary Fig. S2b,c).

### Anosmin-1 enhances endothelial cell migration, proliferation, and tube formation

**Figure 2:**
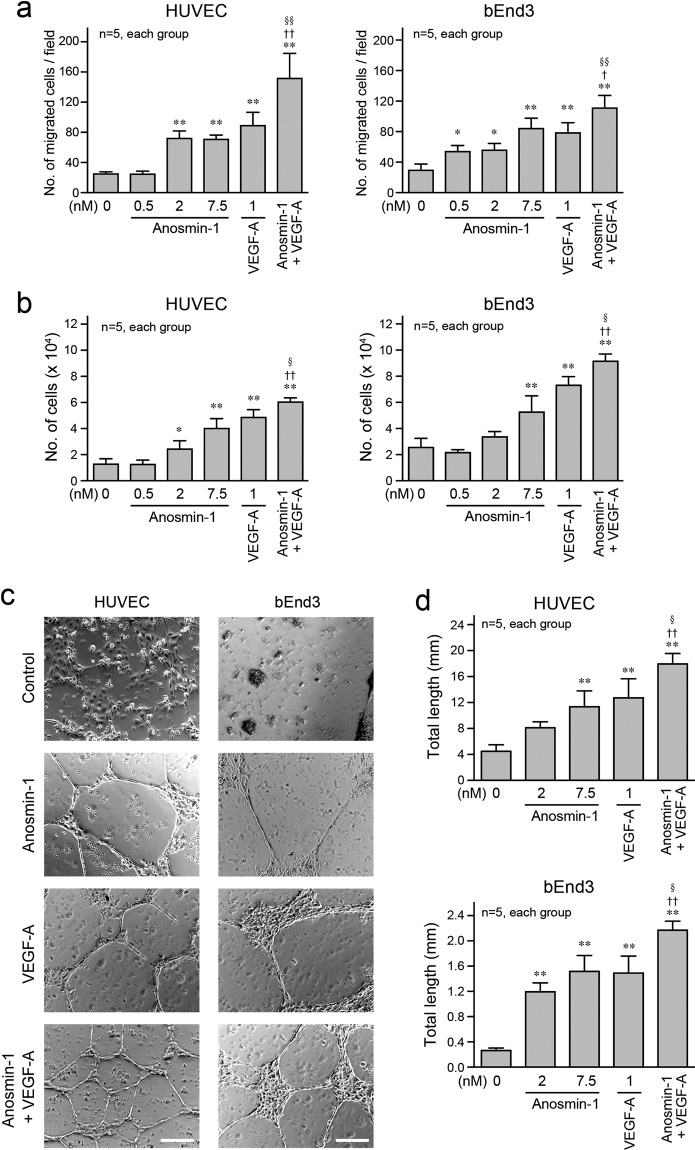
Angiogenic effects of anosmin-1 in endothelial cells. (**a**) Transwell cell migration assay. HUVECs or bEnd3 cells were seeded in the upper compartment, and were treated with the indicated concentrations of anosmin-1, VEGF-A, both 7.5 nM anosmin-1 and 1 nM VEGF-A (Anosmin-1 + VEGF-A), or without reagents (0 nM). The cells that migrated through the Transwell membrane and attached on the underside of the membrane were counted in five different microscopic fields and quantified. (**b**) Cell proliferation assay. Starved HUVECs or bEnd3 cells were treated with the indicated concentrations of anosmin-1, VEGF-A, both 7.5 nM anosmin-1 and 1 nM VEGF-A (Anosmin-1 + VEGF-A), or without reagents (0 nM). After the incubation, the number of cells in the culture dishes was counted. (**c**) Representative images of the 2D tube formation assay. Cells were seeded on a 24-well plate coated with Matrigel, and then, 7.5 nM anosmin-1, 1 nM VEGF-A or both 7.5 nM anosmin-1 and 1 nM VEGF-A (Anosmin-1 + VEGF-A) were added. As a negative control (Control), cells were incubated without reagents. After the incubation and fixation, the formed tubes were observed by light microscopy. Scale bar: 200 μm. (**d**) Summary graphs of (**c**). Total length of the formed tubes was measured. *<0.05 and **P < 0.01 vs. without treatment (0 nM); ^†^P < 0.05 and ^††^P < 0.01 vs. 7.5 nM anosmin-1; ^§^P < 0.05 and ^§§^P < 0.01 vs. 1 nM VEGF-A.

We next investigated the angiogenic activity of anosmin-1 at the cellular level. In the Transwell cell migration assay, treatment with anosmin-1 significantly increased the number of cells that migrated into the lower chamber in two types of endothelial cells, human umbilical vein endothelial cells (HUVECs) and mouse brain endothelioma bEnd3 cells, similar to that with VEGF-A (Fig. 2a). Anosmin-1 as well as VEGF-A also promoted the cell proliferation and two-dimensional (2D) tube formation in these endothelial cells (Fig. 2b–d). These anosmin-1-induced angiogenic effects in endothelial cells were further enhanced by additional treatment with VEGF-A (Fig. 2a–d, Anosmin-1 + VEGF-A), and the effects were greater than those upon treatment with VEGF-A alone. Similarly, when HUVECs were treated with VEGF-A in a constant concentration (1 nM), the cell migration was increased by additional anosmin-1 treatment (0.5–7.5 nM) in a dose-dependent manner (Supplementary Fig. S3a). These results suggest that anosmin-1 works in concert with VEGF-A to promote the angiogenic function of endothelial cells.

### Anosmin-1 activates VEGFR2 by binding to this receptor

**Figure 3:**
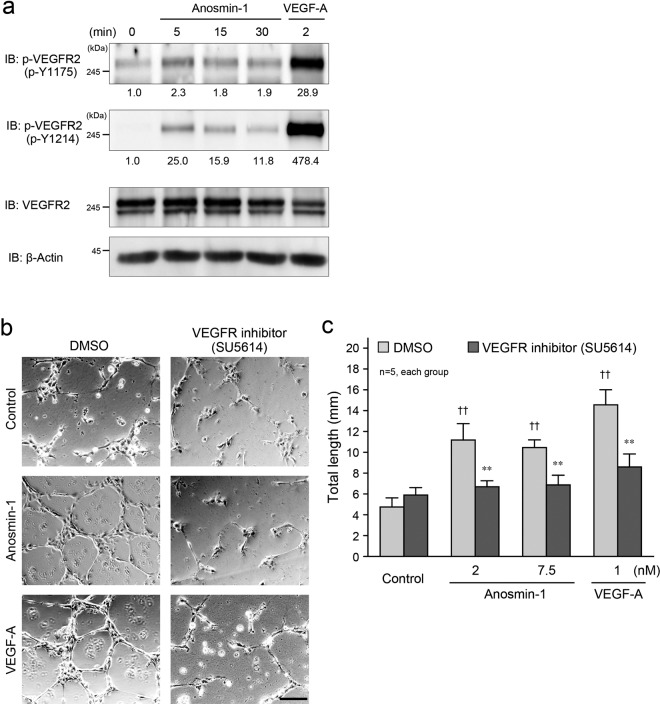
Anosmin-1-induced activation of VEGFR2 for tube formation. (**a**) Phosphorylation of VEGFR2 by anosmin-1 treatment. Starved HUVECs were stimulated with 7.5 nM anosmin-1 for the indicated durations or with 1 nM VEGF-A for 2 min. Cell lysates were analyzed by western blotting with the indicated Abs. The density of each phosphorylated VEGFR2 band normalized by the VEGFR2 band was measured, and the density relative to the value without treatment (0 min) set as 1.0 was calculated and is shown below the band. (**b**) Suppression of anosmin-1-induced tube formation by VEGFR2 inhibitor. HUVECs were seeded onto a Matrigel-coated 24-well plate, and then, 7.5 nM anosmin-1 or 1 nM VEGF-A was added with the VEGFR2 inhibitor (10 μM SU5614) or 0.1% DMSO at the same time. As a negative control (Control), cells were incubated without reagents in the presence of the VEGFR2 inhibitor or 0.1% DMSO. After the incubation and fixation, the formed tubes were observed by light microscopy. Scale bar: 500 μm. (**c**) Summary graphs of (**b**). Total length of the formed tubes was measured. **P < 0.01 vs. DMSO; ^††^P < 0.01 vs. Control.

To explore the underlying molecular mechanism by which anosmin-1 enhanced angiogenesis, the mode of association of anosmin-1 with VEGFR2 was examined. We identified that anosmin-1 induced the phosphorylation (activation) of VEGFR2 in HUVECs, although it was weaker than the VEGF-A-induced phosphorylation of the receptor (Fig. 3a). We also found that the anosmin-1-mediated tube formation in HUVECs was dependent on the activation of VEGFR2, because a VEGFR2 inhibitor, SU5614^23^, significantly impaired the tube formation, compared with dimethyl sulfoxide (DMSO; Fig. 3b,c). In the control conditions using phosphate-buffered saline (PBS; Fig. 2c) and DMSO (Fig. 3b) for the tube formation assay, the cell viability was almost identical and approximately 90% (Supplementary Fig. S3b,c). The inhibitory effect of SU5614 on other receptor tyrosine kinases, such as fibroblast growth factor receptor (FGFR), was also examined, because anosmin-1 has been reported to bind to FGFR1^24^. SU5614 did not inhibit the FGFR phosphorylation induced by the EGM-2 treatment in HUVECs (Supplementary Fig. S3d). We further performed the experiments using HUVECs by knocking down the VEGFR2 expression (Supplementary Fig. S3e). In VEGFR2-knockdown cells, treatment with anosmin-1 as well as VEGF-A did not induce cell migratory activity, compared with scramble RNA-transfected control cells (Supplementary Fig. S3f). These results suggest that anosmin-1 activates VEGFR2 for endothelial tube formation and angiogenesis.

**Figure 4:**
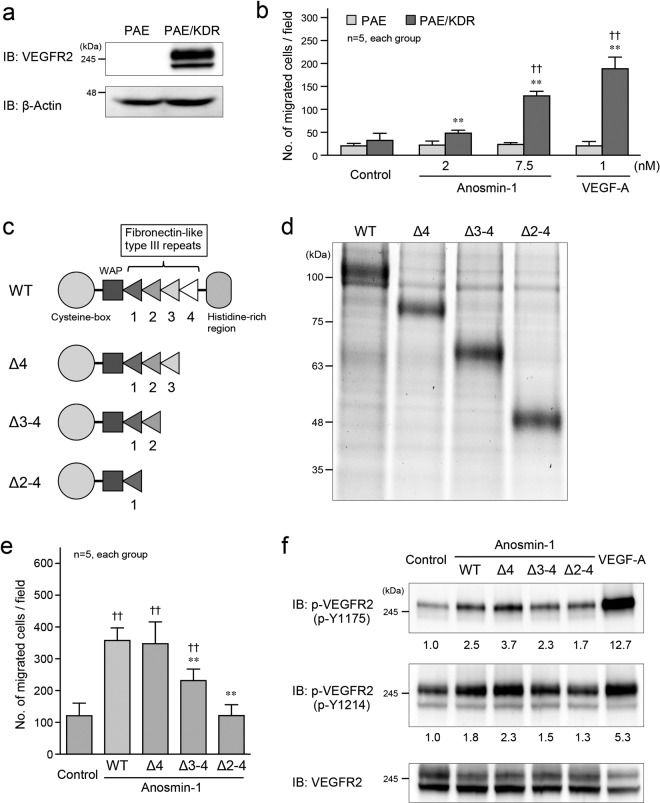
Effects of anosmin-1 deletion mutants on VEGFR2 activation and cell migration. (**a**) Expression of VEGFR2 in PAE and PAE/KDR cells. Cell lysates were analyzed by western blotting with the anti-VEGFR2 and anti-β-actin Abs. (**b**) Transwell cell migration assay. PAE or PAE/KDR cells were seeded in the upper compartment, and were treated with the indicated concentrations of anosmin-1 or VEGF-A, or without anosmin-1 (Control). The cells that moved into the lower chamber were counted in five different microscopic fields. **P < 0.01 vs. PAE; ^††^P < 0.01 vs. Control. (**c**) Schematic structures of anosmin-1 deletion mutants. WAP: whey acidic protein-like domain. (**d**) Generation of recombinant proteins of anosmin-1 WT and mutants. The purified recombinant proteins (0.5 μg each) were used for SDS-PAGE and visualized by SyproRuby staining. (**e**) Transwell cell migration assay. PAE/KDR cells (5.0 × 104 cells/well) were seeded in the upper compartment, and were stimulated with 7.5 nM anosmin-1 WT or the indicated deletion mutants, or without anosmin-1 (Control). The cells that moved into the lower chamber were counted in five different microscopic fields. **P < 0.01 vs. WT; ^††^P < 0.01 vs. Control. (**f**) Phosphorylation of VEGFR2 by treatment with anosmin-1 deletion mutants. Starved PAE/KDR cells were treated with 7.5 nM anosmin-1 WT or the indicated deletion mutants for 5 min or with 1 nM VEGF-A for 2 min. As a negative control (Control), cells were incubated without reagents. Cell lysates were analyzed by western blotting with the indicated Abs. The density of each phosphorylated VEGFR2 band normalized by the VEGFR2 band was measured, and the density relative to the value without treatment (0 min) set as 1.0 was calculated and is shown below the band.

Further, we confirmed the necessity of VEGFR2 for the function of anosmin-1 using another type of endothelial cells, porcine aortic endothelial (PAE) cells. Overexpression of VEGFR2 (KDR) promoted cell migration in response to the treatment with anosmin-1 as well as VEGF-A (Fig. 4a,b). The expression level of FGFR1 was hardly detectable in PAE and PAE/KDR cells compared with HUVEC (Supplementary Fig. S4a) as reported previously^25^. We investigated the domains of anosmin-1 that promote cell migration and activate VEGFR2. Several deletion mutants of anosmin-1 were generated for this purpose (Fig. 4c,d). The Δ4 mutant, which lacks the fourth fibronectin-like type III repeat and the histidine-rich region, showed increased cell migration and VEGFR2 phosphorylation similar to anosmin-1 wild-type (WT), but the other mutants, Δ3–4 and Δ2–4, failed to do so (Fig. 4e,f). These results suggest that the fourth fibronectin-like type III repeat and histidine-rich region were not required for the anosmin-1-induced activation of VEGFR2 and endothelial cell migration. In addition, we wanted to test if the mutants of anosmin-1 related to KS also affect VEGF signaling. To do this, we examined whether mutations at Q131H, C134G and C163R in the WAP domain of anosmin-1 affect the migratory activity. These mutations have been reported to be observed in patients with KS^6^. We found that similar to anosmin-1 WT, all three mutants induced migratory activities in PAE/KDR cells (Supplementary Fig. S4b,c), suggesting that the WAP domain, at least the sites of point mutations, may not contribute to the anosmin-1-mediated cell migration.

**Figure 5:**
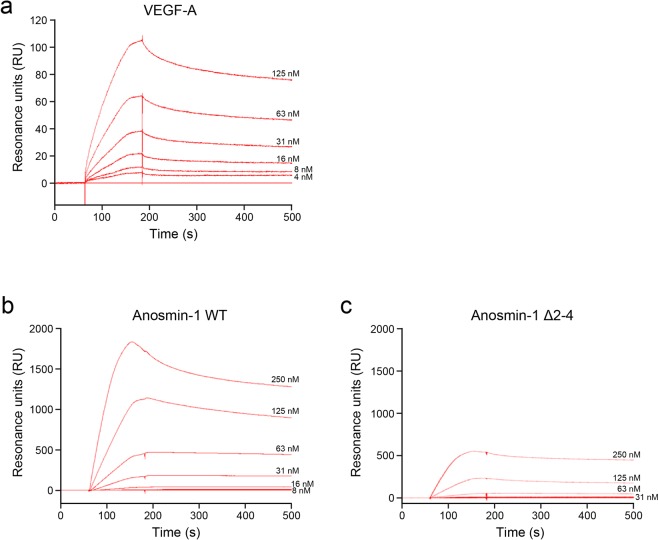
BIAcore analysis for binding to VEGFR2. The data after the injection of VEGF-A (**a**), anosmin-1 WT (**b**), and anosmin-1 ∆2–4 **(c)** at the indicated concentrations are shown.

The above findings led us to hypothesize that anosmin-1 directly interacts with VEGFR2. This was elucidated by a BIAcore binding analysis. As a positive control for the analysis, the binding affinity of VEGF-A to VEGFR2 was examined, and the resonance units (RU) indicating VEGF-A bound to VEGFR2 efficiently cross-linked to the sensor chip showed a remarkable increase by depending on the concentrations of VEGF-A (Fig. 5a). Similarly, the increase in RU was observed between anosmin-1 WT and VEGFR2 in an anosmin-1 dose-dependent manner (Fig. 5b). In contrast to anosmin-1 WT, the RU of the anosmin-1 Δ2–4 mutant to VEGFR2 was markedly decreased (Fig. 5c), indicating the low binding affinity of the mutant to VEGFR2. The dissociation constant (*K*_d_) calculated by the BIAcore analysis for the binding of anosmin-1 WT to VEGFR2 was similar to that of VEGF-A to VEGFR2 (Table 1), suggesting the high affinity of anosmin-1 for its binding to VEGFR2. In contrast, based on the *K*_d_ data, anosmin-1 Δ2–4 mutant is not likely to interact with VEGFR2. Anosmin-1 is reported to liberate extracellular matrix (ECM)-bound FGF and to facilitate its diffusion^6^, which suggests that it might also affect VEGF diffusion by the interaction of anosmin-1 with VEGF. To test this hypothesis, we used the BIAcore analysis to check the binding affinity of anosmin-1 to VEGF-A. Even though the concentration of anosmin-1 WT was increased, the RU values were extremely low (less than 70), compared with those of anosmin-1 WT to VEGFR2 (Supplementary Fig. S5), suggesting that the direct interaction of anosmin-1 with VEGF-A is negative.

**Table 1:** BIAcore analysis of binding of anosmin-1 or VEGF-A to VEGFR2.

	*k*_on_ (M^−1^s^−1^)	*k*_off_ (s^−1^)	*K*_d_ (nM)
VEGF-A	7.3 × 10^4^	1.1 × 10^−3^	15
Anosmin-1 WT	3.5 × 10^4^	1.1 × 10^−3^	32
Anosmin-1 Δ2–4	3.4 × 10^4^	4.6 × 10^−2^	1400

### Anosmin-1 activates the PLC–PKC pathway downstream of VEGFR2

**Figure 6:**
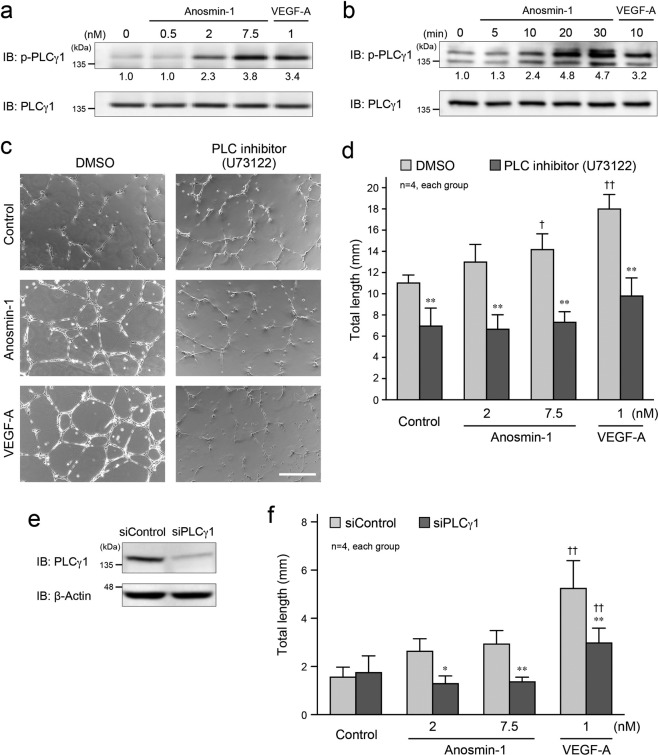
Anosmin-1-induced activation of PLCγ1 and its involvement in tube formation. (**a,b**) Phosphorylation of PLCγ1 by anosmin-1. (**a**) Starved HUVECs were treated with the indicated concentration of anosmin-1 or VEGF-A, or without reagents (0 nM), for 5 min. (**b**) Starved bEnd3 cells were treated with 0.5 nM anosmin-1 or 1 nM VEGF-A for the indicated durations. After the treatment, cells were lysed and analyzed by western blotting with the anti-phospho-PLCγ1 and anti-PLCγ1 Abs. The density of each phosphorylated PLCγ1 band normalized by the PLCγ1 band was measured, and the density relative to the value without treatment (0 nM in **a** or 0 min in **b**) set as 1.0 was calculated and is shown below the band. (**c**) Suppression of the anosmin-1-induced tube formation by the PLC inhibitor. HUVECs were seeded onto a Matrigel-coated 24-well plate and were treated with 7.5 nM anosmin-1 or 1 nM VEGF-A, or without reagents (Control). At the same time, PLC inhibitor (10 μM U73122) or 0.1% DMSO was added. After the incubation and fixation, the formed tubes were observed by light microscopy. Scale bar: 500 μm. (**d**) Summary graph of (**c**). Total length of the formed tubes was quantified. **P < 0.01 vs. DMSO; ^†^P < 0.05 and ^††^P < 0.01 vs. Control. (**e**) Knockdown of PLCγ1. HUVECs were incubated with 10 nM siRNA against PLCγ1 (siPLCγ1) or control siRNA (siControl) for 2 days. Cell lysates were analyzed by western blotting with the anti-PLCγ1 and anti-β-actin Abs. **(f)** Results of the 2D tube formation assay by knockdown of PLCγ1. The assay was performed as indicated in **(c)** in the presence of 10 nM siPLCγ1 or siControl. *P < 0.05 and **P < 0.01 vs. siControl; ^††^P < 0.01 vs. Control.

We further examined which signaling molecules downstream of VEGFR2 were activated by anosmin-1 in endothelial cells and contributed to the anosmin-1-induced angiogenic properties. PLCγ1 is one of the major signaling molecules and can bind to phosphorylated VEGFR2, which leads to the VEGFR2-mediated activation of the intracellular signaling pathway^26,27^. We actually identified the phosphorylation (activation) of PLCγ1 by treatment with anosmin-1 in endothelial cells in concentration- and time-dependent manners (Fig. 6a,b). Next, when the PLC inhibitor, U73122^28^, was applied to the 2D tube formation assay, we found that the anosmin-1- or VEGF-A-promoted tube formation was canceled (Fig. 6c,d). We also observed similar results upon the knockdown of PLCγ1 (Fig. 6e,f). Thus, these results suggest that anosmin-1 stimulates the major downstream signaling of VEGFR2, such as PLCγ1.

**Figure 7:**
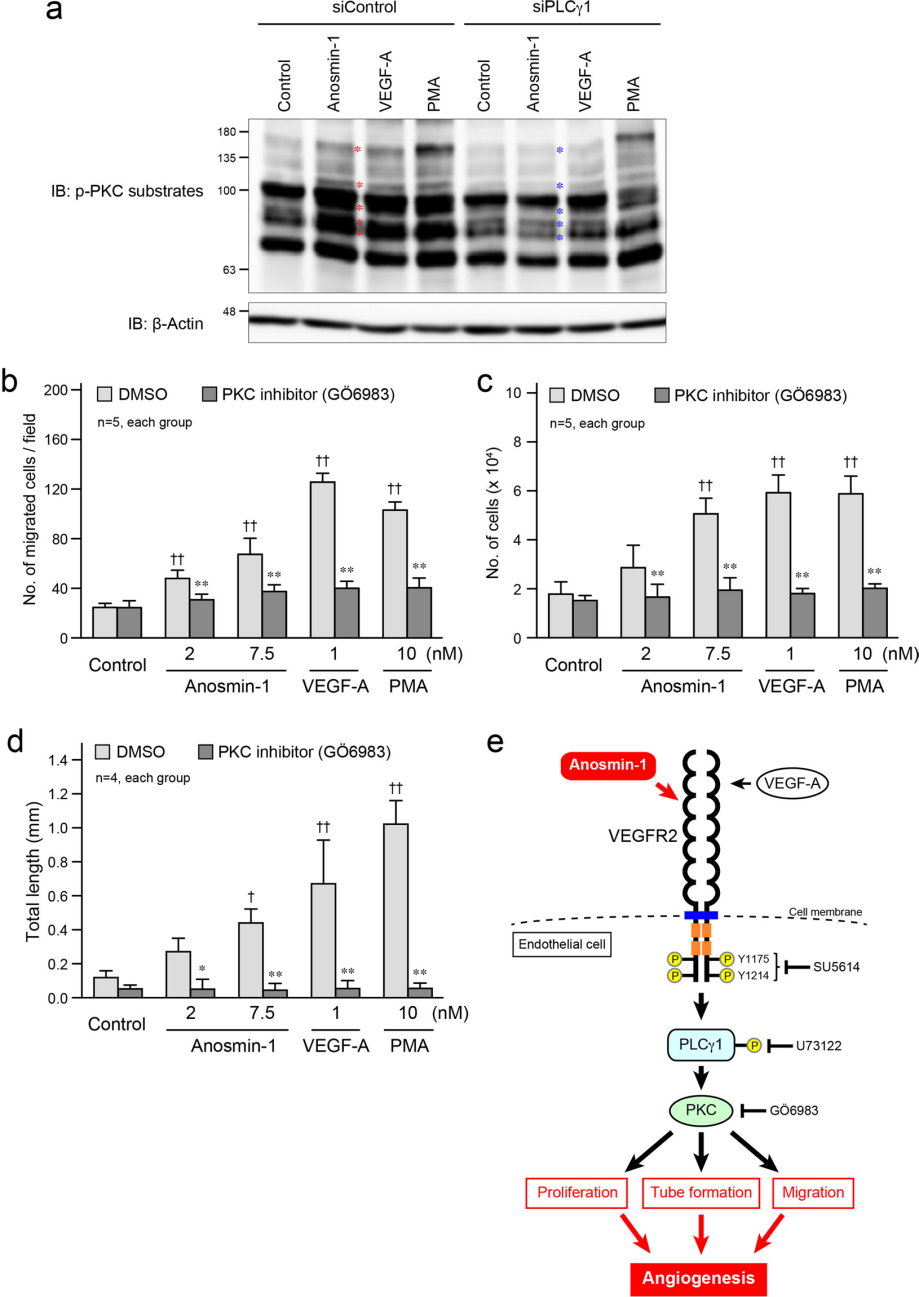
Anosmin-1-induced activation of PKC and its involvement in tube formation. (**a**) Phosphorylation of PKC substrates by anosmin-1. After incubation with 10 nM siPLCγ1 or siControl for 2 days, starved HUVECs were treated with 7.5 nM anosmin-1, 1 nM VEGF-A, 10 nM PMA, or without reagents (Control) for 10 min. PMA was used as a positive control for the PKC activator. Cell lysates were analyzed by western blotting with the anti-phospho-PKC substrates and anti-β-actin Abs. Red asterisks indicate the bands for which the density was increased by anosmin-1 treatment, compared with those in the control lane. Blue asterisks indicate the bands for which the density was attenuated, compared with those of the siControl-treated samples. (**b–d**) Suppression of the anosmin-1-induced cell migration (**b**), proliferation **(c)**, and tube formation (**d**) by the PKC inhibitor. (**b**) HUVECs were seeded in the upper compartment and were treated with the indicated concentrations of anosmin-1, VEGF-A, or PMA in the presence of PKC inhibitor (10 μM GÖ6983) or 0.1% DMSO. The cells that moved into the lower chamber were counted in five different microscopic fields. (**c**) Starved HUVECs were treated with the indicated concentrations of anosmin-1, VEGF-A, or PMA in the presence of PKC inhibitor (10 μM GÖ6983) or 0.1% DMSO. After the incubation, the number of cells in the culture dishes was counted. **(d)** bEnd3 cells were seeded onto a Matrigel-coated 24-well plate, and were treated with the indicated concentrations of anosmin-1, VEGF-A, or PMA. At the same time, PKC inhibitor (10 μM GÖ6983) or 0.1% DMSO was added. Total length of the formed tubes was quantified and is summarized in the graph. *P < 0.05 and **P < 0.01 vs. DMSO; ^†^P < 0.05 and ^††^P < 0.01 vs. Control. (**e**) Schematic representation of this study. Anosmin**-**1 directly binds to VEGFR2 and activates it. Then, the PLCγ1–PKC pathway is activated in anosmin-1-induced angiogenesis.

PLCγ1 activated by VEGFR2 mainly uses PKC for transducing molecular signaling^27^. Treatment with anosmin-1 as well as VEGF-A increased the phosphorylation levels of PKC substrates in endothelial cells (Fig. 7a, the bands marked with red asterisks), and this increase was blocked by the knockdown of PLCγ1 (Fig. 7a, the bands marked with blue asterisks), suggesting the PLCγ1-mediated activation of PKC by anosmin-1. Consistent with this, a PKC inhibitor, GÖ6983^29^, significantly suppressed the anosmin-1-induced cell migration, proliferation, and tube formation in endothelial cells (Fig. 7b–d). Taking these findings together, we may conclude that anosmin-1 binds to VEGFR2 to activate the receptor and its downstream signaling molecules, PLCγ1 and PKC, for enhancing endothelial cell migration, proliferation, and tube formation, resulting in the promotion of angiogenesis (Fig. 7e).

## Discussion

In addition to previous reports describing that anosmin-1 regulates neuronal cell functions, such as migration, branching, and neurite outgrowth, through activation of FGFR1^30–32^, we newly demonstrated in this study that the protein localized at the vessel-like structures in the inner area of chick OB and promoted angiogenesis. The angiogenic characteristics have been considered to be based on anosmin-1-induced promotion of endothelial cell migration, proliferation, and tube formation as shown in this study. Because angiogenic activities of endothelial cells are usually controlled by VEGFR-related signaling^20,33,34^, we focused on the association of anosmin-1 and VEGFR, and successfully identified the involvement of their direct binding in the activation of the receptor-dependent intracellular signaling. Although it has already been shown by another research group that anosmin-1 also directly binds to another growth factor receptor, FGFR1^24^, for the promotion of migration in neuroblast cells, this is the first study to reveal the interaction between anosmin-1 and VEGFR2 and novel functions of anosmin-1 in the angiogenic activity. The molecular affinity of anosmin-1 with VEGFR2 was similar to that of VEGF-A with VEGFR2; therefore, anosmin-1 might be a novel ligand for VEGFR2.

Through further experiments to examine the mode of interaction of anosmin-1 and VEGFR2, we showed that the anosmin-1 N-terminal domains including the cysteine-box domain, WAP, and three fibronectin-like type III repeats were necessary for the activation of VEGFR2. This mode of interaction slightly differs from the anosmin-1–FGFR1 binding, which requires only the first fibronectin-like type III repeat in addition to the cysteine-box domain and WAP^24^. We successfully showed that anosmin-1 additively enhanced the VEGF-A-induced endothelial angiogenic activities. However, the details of how both anosmin-1 and VEGF-A cooperatively facilitate the activities through VEGFR2 have not been revealed in this study. Although it was confirmed that VEGF-A binds to VEGFR2 at the second, third, and fourth loops of the immunoglobulin-like domains^35^, the anosmin-1-binding site in VEGFR2 has not been determined yet, but might differ from the VEGF-A-binding site because of their additive roles in the angiogenic activities. These issues should be clarified in future work.

Similar to VEGFR2, several molecules have been reported to interact with anosmin-1. In the FGF–FGFR signaling, anosmin-1 directly binds to FGFR1 as described above^24^. By this binding, anosmin-1 regulates the cell migration of olfactory neuroblast FNC-B4 cells in the bimodal mode. The chemoattractant or chemorepellent activity of anosmin-1 together with FGFR1 in the cells was dependent on the concentration of anosmin-1. This may contribute to the optimal adjustment of migration of neuronal cells during the formation of OB. The first fibronectin-like type III repeat and the WAP domain are essential for the binding of anosmin-1 to FGFR1, and these regions plus N-terminal cysteine-box are sufficient to induce GnRH neuroblast migration^24^. KS patients with mutations, such as at Q131H, C134G and C163R in the WAP domain, have typical symptoms of KS, which are probably caused by affecting the migration of OB neurons in humans. The anosmin-1-induced FGFR signaling is important for the proper formation of OB. In addition, we showed that anosmin-1 directly interacted with VEGFR2 and that the interaction promoted the activation of signaling pathways downstream of VEGFR2 in endothelial cells. These findings may proceed the research to reveal the biological property of anosmin-1 in the OB angiogenesis. Other KS patients have been reported to have the deletion mutant in the second fibronectin-like type III repeat (e.g. E320X, Y328X)^6^, which is critical for the anosmin-1–VEGFR2 binding. This may support the importance of the VEGFR2 binding region of anosmin-1 in KS, although currently we could not identify the patients with the defect in the *ANOS1* gene who suffered from vascular abnormalities related to the anosmin-1-mediated VEGFR2 signaling. Anosmin-1 also binds to FGF, WNT, and BMP^22^. These bindings are essential for cranial neural crest formation. Laminin and fibronectin are also binding partners of anosmin-1 via its first and third fibronectin-like type III domains^36^. The molecular bindings may regulate the cell–cell and cell–ECM interactions and the anosmin-1-induced chemoattraction of neuronal precursors. Urokinase-type plasminogen activator (uPA) uses anosmin-1 to enhance its enzymatic activity by binding to the protein^37^. Anosmin-1 also directly interacts with heparan sulfate for the cell surface localization of the anosmin-1–uPA complex that promotes cell proliferation of prostate cancer cells.

The appropriate development of tissues and organs during embryogenesis is heavily dependent on a sufficient supply of nutrients and oxygenation^38,39^. These processes are primarily accomplished by the vascular system. The normal formation of a functional vascular system is one of the most critical and earliest events that occur during embryogenesis^40,41^. Several pro-angiogenic factors, such as FGF8^42,43^, VEGF-A^44^, VEGF-C^45^, and prokineticin (PROK)^46^, are expressed in the OB during the development and are involved in the formation of OB by facilitating the neuronal cell proliferation and migration. For example, the downregulation of PROK2 (or its receptor) and VEGF-A in mice showed OB defects. VEGF-A depletion in the brain of mice exhibited the impaired vascular phenotype with the decreased size of OB. Due to the diameter of enlarged OB reaching approximately 1 mm in E10 chick embryos, the angiogenic events may be highly necessary to sustain the organ to maintain neuronal cells, such as olfactory and GnRH neurons. Thus, in turn, if the angiogenic process during the development of OB in the embryo is impaired even slightly, this might affect development. In this context, the existence and role of anosmin-1 in the promotion of angiogenesis in OB are likely to be required, in addition to those of VEGF and other pro-angiogenic factors.

Besides the *ANOS1* gene, several causative genes for KS have been identified, such as *FGFR1*, *PROK2*, and *PROKR2*^47,48^. As described above, FGFR1 is a binding partner of anosmin-1, and has important roles in both physiological and pathological angiogenesis^49,50^. Under physiological conditions, FGFR1 and VEGFR2 are used for vessel formation in the embryo. PROK2, also named Bv8, is a ligand for a G protein-coupled receptor, PROKR2. Recently, it has been found that the PROK2–PROKR2 pathway is crucial for angiogenesis in embryonic development and that the expression of PROK2 is regulated by the TBX20 transcription factor^51^. Considering that the products of genes causative of KS contribute to embryonic angiogenesis, abnormality in the angiogenic process appears to be important for the pathogenesis of KS.

By searching the database of Mouse Genome Informatics, we found that *Wfdc18* and *Slamf6* are listed as the mouse *Kal1* genes, which may be related to *ANOS1*. However, the Wfdc18 protein consists of only 74 amino acids (aa), while human anosmin-1 has 680 aa. The protein possesses the WAP domain alone, and the homology to anosmin-1 is merely 32% in the domain. Slamf6 consists of 331 aa, which is about a half of anosmin-1, without any domains existing in anosmin-1. The homology to anosmin-1 is very low (18%). Thus, these genes are not likely to be the orthologs of *ANOS1*.

As for the intracellular signaling for the anosmin-1-induced activation of VEGFR2, we identified PLCγ1 and PKC as important molecules for the enhancement of cell migration, proliferation, and tube formation in endothelial cells treated with anosmin-1. The sites in VEGFR2 phosphorylated by anosmin-1 were Y1175 and Y1214. When these sites were phosphorylated, many signaling molecules, such as Shb, Sck, and Nck^33^, are recruited and bind to the phospho-tyrosine residues, resulting in the activation of these molecules and transduction of the signals to downstream molecules. We cannot completely rule out the possibility that, in addition to the PLCγ1–PKC pathway, other pathways may be activated in endothelial cells upon treatment with anosmin-1 through VEGFR2 and promote angiogenesis. However, based on the results that the PLCγ1 and PKC inhibitors almost entirely block the anosmin-1-induced angiogenic characteristics, it is considered that PLCγ1 and PKC are the main players that function downstream of VEGFR2 when the receptor is activated by anosmin-1. Furthermore, there might be unidentified cell surface molecules that can interact with anosmin-1 and induce angiogenesis. Future work should aim at discovering such molecules, which may also be useful for obtaining a deeper understanding of KS.

In conclusion, this study showed that anosmin-1 induced signal transduction for angiogenesis via VEGFR2 in endothelial cells, and could play an important role in OB development during embryogenesis. These results may have clinical implications via the development or improvement of therapeutic applications for KS.

## Experimental Procedures

### Cell culture

HUVECs were purchased from Lonza (Basel, Switzerland) and maintained in EGM-2 media^52^. bEnd3 cells were purchased from American Type Culture Collection (Manassas, VA, USA) and cultured in Dulbecco’s Modified Eagle Medium (DMEM) supplemented with 10% fetal bovine serum (FBS)^53^. PAE cells and PAE/KDR cells were provided by Dr. Michael Klagsbrun and cultured in Ham’s F-12 supplemented with 10% FBS^54^.

### Reagents and siRNAs

Recombinant human anosmin-1, its deletion mutants, and mutants with a point mutation at Q131H, C134G and C163R in the WAP domain were purified by ourselves. Briefly, cDNAs of anosmin-1 WT or its mutants were inserted into the pSecTag2 mammalian expression vector (Thermo Fisher Scientific, Waltham, MA, USA) to generate the recombinant proteins in 293 T cells. The collected conditioned media were applied to the HisTrap HP column (GE Healthcare, Piscataway, NJ, USA) and PD-10 column (GE Healthcare) to purify the proteins. The concentrations of purified proteins were measured using the DC protein assay kit (Bio-Rad, Hercules, CA, USA). Recombinant chick anosmin-1 was generated and purified as described above. Recombinant human VEGF-A_165_ was purchased from R&D Systems (Minneapolis, MN, USA), the VEGFR2 kinase inhibitor SU5614 from Merck (Whitehouse Station, NJ, USA), the PLC inhibitor U73122 from Abcam (Cambridge, UK), the PKC inhibitor GÖ6983 from Wako Pure Chemical (Osaka, Japan), and phorbol 12-myrisatate 13-acetate (PMA) from Wako Pure Chemical. siGENOME smart pool control siRNA (D-001206) and PLCγ1 siRNA (M-040978) were purchased from Thermo Fisher Scientific. For knockdown of VEGFR2, the following siRNA was used: 5′-GGGCUUUACUAUUCCCAGC-3′. The scramble RNA used for the control was as follows: 5′-CAGUCGCGUUUGCGACUGG-3′.

### Antibodies

An anti-chick anosmin-1 rabbit pAb was provided by Dr. Kenneth M. Yamada. The following primary Abs were purchased from commercial companies: an anti-β-actin (clone 8H10D10) mouse mAb, an anti-VEGF Receptor 2 (clone 55B11) rabbit mAb, an anti-phospho-VEGFR2 (Y1175) (clone 19A10) rabbit mAb, an anti-FGFR1 (clone D8E4) rabbit mAb, an anti-phospho-FGF Receptor (Tyr653/654) rabbit pAb, an anti-phospho-PKC substrate rabbit pAb, and an anti-phospho-PLCγ1 (Y783) rabbit pAb from Cell Signaling Technology (Danvers, MA, USA); an anti-PLCγ1 (clone B-4) mouse mAb and an anti-Flk-1 rabbit pAb from Santa Cruz Biotechnology (Dallas, TX, USA); an anti-CD31 (PECAM-1) (clone MEC 13.3) rat mAb from BD Pharmingen (Franklin Lakes, NJ, USA); an anti-phospho-VEGFR2 (Y1214) rabbit pAb from St John’s Laboratory (London, UK); and an anti-GAPDH (clone 3H12) mouse mAb from Medical & Biological Laboratories (Nagoya, Japan).

The following secondary Abs were used: horseradish peroxidase (HRP)-conjugated anti-rabbit IgG and HRP-conjugated anti-mouse IgG Abs from Jackson ImmunoResearch (West Grove, PA, USA). Alexa Fluor 594 anti-rat IgG, Alexa Fluor 488 anti-rabbit IgG, and Alexa Fluor 488 Phalloidin were purchased from Thermo Fisher Scientific.

### *In situ* hybridization

The tissue samples were prepared for this experiment as described previously^55^. Briefly, we used chick full-length anosmin-1 mRNA for the anosmin-1 RNA probe, which was labeled with digoxigenin (Roche Diagnostics, Basel, Switzerland). The samples were incubated with the probe at 65 °C for 18 h. After washing with TTBST (Tris-buffered saline containing 0.1% Tween-20 and 0.5% Triton X-100 [Wako Pure Chemical]), the samples were then incubated with anti-digoxigenin-AP antibody (Roche) in TTBST including 5% horse serum (Thermo Fisher Scientific) at 4 °C overnight. The *in situ* color reactions were developed in a mixture of 5-bromo-4-chloro-3-indolylphosphate *p*-toluidine salt and nitro blue tetrazolium chloride solutions (Nacalai Tesque, Kyoto, Japan) in color reaction buffer (50 mM Tris-HCl at pH 9.5, 150 mM NaCl, 25 mM MgCl_2_) at room temperature. Stained sections were imaged under a microscope (ECLIPSE Ti-U; Nikon, Tokyo Japan) and the images were analyzed with NIS-Elements BR Analysis software (Nikon).

### Immunohistochemical analysis

This analysis was performed as described previously^22^. The sectioned samples were incubated with anti-anosmin-1 and anti-CD31 primary Abs at 4 °C for 1 day, followed by incubation for 1 h with secondary Abs of Alexa Fluor 594 anti-rat IgG for CD31 and of Alexa Fluor 488 anti-rabbit IgG for anosmin-1. The stained samples were observed using a Leica SP8 confocal microscope.

### Tissue culture of pulmonary artery and OB of chick embryo

Fertilized eggs were purchased from Shiroyama Keien (Sagamihara, Japan) or Yamagishi internet store (Yotsukaichi, Japan), and incubated for 10–12 days at 37 °C. The OB and pulmonary artery were isolated from the embryos and cut into 0.4-mm fragments. Chamber slides were prefilled with 3 µL of Matrigel. The tissue fragment was put in each well and covered in 10 µL of Matrigel. For the siRNA treatment, EBM-2 (200 µL) supplemented with 2% FBS was added after the Matrigel had solidified, and then, siRNAs (scramble 5′-CAGUCGCGUUUGCGACUGG-3′ for the control; chick anosmin-1 siRNA #1 5′-GGACUGGUAGAUCCUUACC-3′ and chick anosmin-1 siRNA #2 5′-GCUGUGAAGCUGAUAGUGA-3′ for knockdown of anosmin-1) were further added on OB covered with the Matrigel according to the Matrigel-adapted transfection protocol described previously^56^. For enhancement of vessel formation, the OB and pulmonary artery were additionally treated with anosmin-1 (15 nM for OB, 30 nM for pulmonary artery) and/or EGM-2 (200 µL). The tissue fragments were incubated at 37 °C in a humidified atmosphere with 5% CO_2_. After 3 days, they were washed with serum-free EBM-2 twice, fixed with 4% PFA at room temperature for 15 min, and washed with PBS three times. They were then solubilized by 0.2% Triton-X 100 for 30 min and blocked with 3% BSA in PBS for 20 min, followed by incubation with an anti-mouse CD31 rat mAb diluted with blocking buffer (1/100) at 4 °C for 2 days. These tissues were incubated with Alexa Fluor 594 anti-rat IgG and Alexa Fluor 488 Phalloidin overnight. Mounting medium was finally added and the tissues were enclosed with cover glass. The samples were observed using a Leica SP8 confocal microscope. The experiments using chick embryos and the mouse OB samples were approved by Kyoto Sangyo University ethics committee for animal care, handling, and termination, and the Shiga University of Medical Science Animal Care and Use Committee, respectively.

### Quantitative PCR

To evaluate the expression level of anosmin-1 after the siRNA transfection into OB isolated from chick embryos, quantitative PCR (qPCR) was performed as described previously^57^. mRNA was extracted from OB specimens that were cultured for 3 days as described above after the siRNA transfection. Data were quantified by the standard curve method and were adjusted using the GAPDH mRNA expression level as an internal control. Primer sequences for quantitative PCR experiments were as follows: *Anosmin-1* forward 5′-TTCTCCACCGCCAGATGC-3′ and reverse 5′-CCAGGATTCTTTGCAGGGCTC-3′; *GAPDH* forward 5′-TCAAGGCTGAGAACGGGAAAC-3′ and reverse 5′-TTCCCATTCAGCTCAGGGATG-3′.

### Transwell cell migration assay

Transwell inserts with a pore size of 8.0 µm (Corning, New York City, NY, USA) were used. The experiment was performed as previously described, with some modifications^58^. Membranes were coated with 0.5% gelatin. Cells (HUVECs: 5.0 × 10^4^/well, bEnd3 cells: 1.0 × 10^3^/well, PAE/KDR cells: 5.0 × 10^4^/well) were suspended in EBM-2 supplemented with 0.1% FBS, seeded in the upper compartment, and cultured for 16 h with the indicated concentrations of anosmin-1 or VEGF-A. Migrated cells were stained with Diff-Quick. The stained cells in five microscopic fields were counted.

### Proliferation assay

Cells were seeded in 24-well plates (HUVECs: 1.0 × 10^4^ cells/well, bEnd3 cells: 2.0 × 10^4^ cells/well) and cultivated for 6 h. Then, cultivation medium was replaced (HUVECs: 2% FBS EBM-2, bEnd3 cells: 5% FBS DMEM) and incubated for 24 h for starvation. The indicated concentrations of anosmin-1 or VEGF-A were added to the medium and incubated for 72 h. Cells were washed with PBS and detached with 0.05% trypsin. Cells were counted with Z2 COUNTER Particle Count and Size Analyzer (Beckman Coulter, Brea, CA, USA).

### 2D tube formation assay

A 24-well plate was coated with 25 µL of Matrigel (Matrigel Matrix Basement Membrane Growth Factor Reduced; Corning) and incubated at 37 °C until the Matrigel had solidified. The cell-suspending media (500 µL) were layered onto Matrigel (HUVECs: 1.0 × 10^5^ cells/well in 1% FBS EBM-2, bEnd3 cells: 1.0 × 10^5^ cells/well in 0.5% FBS DMEM) and incubated for 45 min at 37 °C. After 200 µL of medium had been removed, the indicated reagents or chemicals were added, and cells were further incubated at 37 °C for 8 h. Then, cells were fixed with 2% PFA at room temperature for 15 min and washed with PBS twice, followed by microscopic observation (Eclipse Ti-U; Nikon). The total length of the formed tubes per microscopic field was measured by ImageJ software (National Institutes of Health, Bethesda, MD, USA) and quantified from five microscopic fields.

### Phosphorylation analysis

Cells were washed twice with 1 × HBSS, and then, HUVECs and PAE/KDR cells were incubated in 2% FBS EBM-2 and Ham’s F-12 without FBS for 4 h for starvation. The cells were stimulated with 7.5 nM anosmin-1 or 1 nM VEGF-A for the indicated durations and then lysed with RIPA buffer (50 mM Tris-HCl [pH 7.4], 100 mM NaCl, 0.1% SDS, 0.5% sodium deoxycholate, 1% Nonidet P-40) containing 1% phosphatase inhibitor cocktails 2 and 3 (Sigma-Aldrich, St. Louis, MO, USA). After SDS-PAGE, proteins were transferred to a polyvinylidene difluoride membrane (Millipore, Billerica, MA, USA) and blotted with the phospho-specific primary Abs diluted in 5% Blocking One (Nacalai Tesque) in TBST at 4 °C overnight, followed by incubation with the HRP-conjugated secondary Ab. The blots were treated with chemiluminescent substrate solution (Thermo Fisher Scientific) and exposed to LAS-4000 Mini (Fujifilm Co., Tokyo, Japan) to visualize immunoreactive bands.

### BIAcore binding analysis

To analyze the kinetics of the binding of VEGF-A with VEGFR2 and the binding of anosmin-1 with VEGFR2 or VEGF-A, we used a BIAcore surface plasmon resonance system at 20 °C. The biosensor chip CM5 was activated by *N*-ethyl-*N*′-(3-dimethylaminopropyl)-carbodiimide hydrochloride and *N*-hydroxysuccinimide, in accordance with the supplier’s instructions. Recombinant human VEGFR2-Fc (50 µg/mL; BioLegend, San Diego, CA, USA) or VEGF-A (50 µg/mL; R&D systems, Minneapolis, MN) in 10 mM sodium acetate, pH 4.5, was injected onto the activated CM5 chip for 7 min. The remaining active sites on the chip were blocked by 0.5 M ethanolamine. To perform binding assays, samples with various concentrations of anosmin-1, its mutant, or VEGF-A in PBS with 0.35 M NaCl were injected at a flow rate of 30 µL/min. Changes in refractive index upon binding were used for kinetic measurements. The affinity of anosmin-1, its mutant, or VEGF-A binding to VEGFR2 was calculated from the on- and off-rates.

### Cell viability assay

Cells incubated on the Matrigel and treated with 0.1% PBS or 0.1% DMSO for 16 h were stained with 0.5% Trypan Blue (Nacalai Tesque) for 1 min. The number of cells stained in blue (dead cells) or not (live cells) was counted using a IX83 inverted microscope (Olympus, Tokyo, Japan).

### Statistics

Data are expressed as mean ± S.D. Experiments were performed at least three times independently. Statistical significance was determined by one-way or two-way ANOVA, as appropriate. If the results of ANOVA were significant, individual differences were evaluated using the Bonferroni post-test. A value of p < 0.05 was considered to be statistically significant.

## Supplementary information

Supplementary Information

Supplementary Information 2

## References

[1] Soussi-Yanicostas N (1996). Initial characterization of anosmin-1, a putative extracellular matrix protein synthesized by definite neuronal cell populations in the central nervous system. J. Cell Sci..

[2] Soussi-Yanicostas N (2002). Anosmin-1, defective in the X-linked form of Kallmann syndrome, promotes axonal branch formation from olfactory bulb output neurons. Cell.

[3] Gianola S, de Castro F, Rossi F (2009). Anosmin-1 stimulates outgrowth and branching of developing Purkinje axons. Neuroscience.

[4] Yanicostas C, Herbomel E, Dipietromaria A, Soussi-Yanicostas N (2009). Anosmin-1a is required for fasciculation and terminal targeting of olfactory sensory neuron axons in the zebrafish olfactory system. Mol. Cell. Endocrinol..

[5] Bribián A, Barallobre MJ, Soussi-Yanicostas N, de Castro F (2006). Anosmin-1 modulates the FGF-2-dependent migration of oligodendrocyte precursors in the developing optic nerve. Mol. Cell. Neurosci..

[6] Hu Y, Bouloux PM (2011). X-linked GnRH deficiency: role of KAL-1 mutations in GnRH deficiency. Mol. Cell. Endocrinol..

[7] Tickotsky N, Moskovitz M (2014). Renal agenesis in Kallmann syndrome: a network approach. Ann. Hum. Genet..

[8] Franco B (1991). A gene deleted in Kallmann’s syndrome shares homology with neural cell adhesion and axonal path-finding molecules. Nature.

[9] Legouis R (1991). The candidate gene for the X-linked Kallmann syndrome encodes a protein related to adhesion molecules. Cell.

[10] Soussi-Yanicostas N (1998). Anosmin-1 underlying the X chromosome-linked Kallmann syndrome is an adhesion molecule that can modulate neurite growth in a cell-type specific manner. J. Cell Sci..

[11] Legouis R, Lievre CA, Leibovici M, Lapointe F, Petit C (1993). Expression of the KAL gene in multiple neuronal sites during chicken development. Proc. Natl. Acad. Sci. USA.

[12] Ardouin O (2000). Characterization of the two zebrafish orthologues of the KAL-1 gene underlying X chromosome-linked Kallmann syndrome. Mech. Dev..

[13] Andrenacci D (2006). Functional dissection of the Drosophila Kallmann’s syndrome protein DmKal-1. BMC Genet..

[14] Hudson ML, Kinnunen T, Cinar HN, Chisholm AD (2006). C. elegans Kallmann syndrome protein KAL-1 interacts with syndecan and glypican to regulate neuronal cell migrations. Dev. Biol..

[15] Hu Y, Tanriverdi F, MacColl GS, Bouloux PM (2003). Kallmann’s syndrome: molecular pathogenesis. Int. J. Biochem. Cell Biol..

[16] Christian JC, Bixler D, Dexter RN, Donohue JP (1971). Hypogandotropic hypogonadism with anosmia: the Kallmann syndrome. Birth Defects Orig. Artic. Ser..

[17] Olsson AK, Dimberg A, Kreuger J, Claesson-Welsh L (2006). VEGF receptor signalling - in control of vascular function. Nat. Rev. Mol. Cell Biol..

[18] Bovetti S (2007). Blood vessels form a scaffold for neuroblast migration in the adult olfactory bulb. J. Neurosci..

[19] Lazarus A, Keshet E (2011). Vascular endothelial growth factor and vascular homeostasis. Proc. Am. Thorac. Soc..

[20] Shimizu, A., Zankov, D. P., Kurokawa-Seo, M. & Ogita, H. Vascular Endothelial Growth Factor-A Exerts Diverse Cellular Effects via Small G Proteins, Rho and Rap. *Int. J. Mol. Sci*., **19** (2018). 10.3390/ijms19041203 PMC5979568 29659486

[21] Yancopoulos GD (2000). Vascular-specific growth factors and blood vessel formation. Nature.

[22] Endo Y, Ishiwata-Endo H, Yamada KM (2012). Extracellular matrix protein anosmin promotes neural crest formation and regulates FGF, BMP, and WNT activities. Dev. Cell.

[23] Jia H (2004). Vascular endothelial growth factor (VEGF)-D and VEGF-A differentially regulate KDR-mediated signaling and biological function in vascular endothelial cells. J. Biol. Chem..

[24] Hu Y (2009). Novel mechanisms of fibroblast growth factor receptor 1 regulation by extracellular matrix protein anosmin-1. J. Biol. Chem..

[25] Wennström S, Landgren E, Blume-Jensen P, Claesson-Welsh L (1992). The platelet-derived growth factor beta-receptor kinase insert confers specific signaling properties to a chimeric fibroblast growth factor receptor. J. Biol. Chem..

[26] Matsumoto T, Mugishima H (2006). Signal transduction via vascular endothelial growth factor (VEGF) receptors and their roles in atherogenesis. J. Atheroscler. Thromb..

[27] Shibuya M (2013). VEGFR and type-V RTK activation and signaling. Cold Spring Harb. Perspect. Biol..

[28] Hildebrandt JP, Plant TD, Meves H (1997). The effects of bradykinin on K+ currents in NG108-15 cells treated with U73122, a phospholipase C inhibitor, or neomycin. Br. J. Pharmacol..

[29] Kim SY (2011). PKC inhibitors RO 31-8220 and Gö 6983 enhance epinephrine-induced platelet aggregation in catecholamine hypo-responsive platelets by enhancing Akt phosphorylation. BMB Rep..

[30] Díaz-Balzac CA, Lázaro-Peña MI, Ramos-Ortiz GA, Bülow HE (2015). The adhesion molecule KAL-1/anosmin-1 regulates neurite branching through a SAX-7/L1CAM-EGL-15/FGFR receptor complex. Cell Rep..

[31] García-González D (2010). Dynamic roles of FGF-2 and Anosmin-1 in the migration of neuronal precursors from the subventricular zone during pre- and postnatal development. Exp. Neurol..

[32] Hu Y, Poopalasundaram S, Graham A, Bouloux PM (2013). GnRH neuronal migration and olfactory bulb neurite outgrowth are dependent on FGF receptor 1 signaling, specifically via the PI3K p110α isoform in chick embryo. Endocrinology.

[33] Holmes K, Roberts OL, Thomas AM, Cross MJ (2007). Vascular endothelial growth factor receptor-2: structure, function, intracellular signalling and therapeutic inhibition. Cell Signal..

[34] Shibuya M, Claesson-Welsh L (2006). Signal transduction by VEGF receptors in regulation of angiogenesis and lymphangiogenesis. Exp. Cell Res..

[35] Shinkai A (1998). Mapping of the sites involved in ligand association and dissociation at the extracellular domain of the kinase insert domain-containing receptor for vascular endothelial growth factor. J. Biol. Chem..

[36] Murcia-Belmonte V, Esteban PF, García-González D, De Castro F (2010). Biochemical dissection of Anosmin-1 interaction with FGFR1 and components of the extracellular matrix. J. Neurochem..

[37] Hu Y, González-Martínez D, Kim SH, Bouloux PM (2004). Cross-talk of anosmin-1, the protein implicated in X-linked Kallmann’s syndrome, with heparan sulphate and urokinase-type plasminogen activator. Biochem. J..

[38] Clapp JF (2003). The effects of maternal exercise on fetal oxygenation and feto-placental growth. Eur. J. Obstet. Gynecol. Reprod. Biol..

[39] Cross JC (2006). Placental function in development and disease. Reprod. Fertil. Dev..

[40] Charpentier MS, Conlon FL (2014). Cellular and molecular mechanisms underlying blood vessel lumen formation. Bioessays.

[41] Saha MS, Cox EA, Sipe CW (2004). Mechanisms regulating the origins of the vertebrate vascular system. J. Cell. Biochem..

[42] Hébert JM, Lin M, Partanen J, Rossant J, McConnell SK (2003). FGF signaling through FGFR1 is required for olfactory bulb morphogenesis. Development.

[43] Olsen SK (2006). Structural basis by which alternative splicing modulates the organizer activity of FGF8 in the brain. Genes. Dev..

[44] Licht T (2010). VEGF is required for dendritogenesis of newly born olfactory bulb interneurons. Development.

[45] Ward MC, Cunningham AM (2015). Developmental expression of vascular endothelial growth factor receptor 3 and vascular endothelial growth factor C in forebrain. Neuroscience.

[46] Martin C (2011). The role of the prokineticin 2 pathway in human reproduction: evidence from the study of human and murine gene mutations. Endocr. Rev..

[47] Dodé C (2003). Loss-of-function mutations in FGFR1 cause autosomal dominant Kallmann syndrome. Nat. Genet..

[48] Dodé C (2006). Kallmann syndrome: mutations in the genes encoding prokineticin-2 and prokineticin receptor-2. PLoS Genet..

[49] Katoh M, Nakagama H (2014). FGF receptors: cancer biology and therapeutics. Med. Res. Rev..

[50] Murakami M, Simons M (2008). Fibroblast growth factor regulation of neovascularization. Curr. Opin. Hematol..

[51] Meng S (2018). TBX20 regulates angiogenesis through the prokineticin 2-prokineticin receptor 1 pathway. Circulation.

[52] Shimizu A (2008). ABL2/ARG tyrosine kinase mediates SEMA3F-induced RhoA inactivation and cytoskeleton collapse in human glioma cells. J. Biol. Chem..

[53] Brown RC, Morris AP, O’Neil RG (2007). Tight junction protein expression and barrier properties of immortalized mouse brain microvessel endothelial cells. Brain Res..

[54] Kroll J, Waltenberger J (1997). The vascular endothelial growth factor receptor KDR activates multiple signal transduction pathways in porcine aortic endothelial cells. J. Biol. Chem..

[55] Ding L (2005). Short-term lineage analysis of dorsally derived Olig3 cells in the developing spinal cord. Dev. Dyn..

[56] Morgan RG (2018). Optimized delivery of siRNA into 3D tumor spheroid cultures *in situ*. Sci. Rep..

[57] Pang X (2016). Novel Therapeutic Role for Dipeptidyl Peptidase III in the Treatment of Hypertension. Hypertension.

[58] Ahmat Amin MKB (2018). Epithelial membrane protein 1 promotes tumor metastasis by enhancing cell migration via copine-III and Rac1. Oncogene.

