# Supplementary material for: Anosmin-1 activates vascular endothelial growth factor receptor and its related signaling pathway for olfactory bulb angiogenesis
Source: Sci Rep. 2020 Jan 13;10:188. doi: 10.1038/s41598-019-57040-3 (PMC6957483; doi:10.1038/s41598-019-57040-3)
Supplement: Supplementary file 1 — Supplementary Information [file 41598_2019_57040_MOESM1_ESM.pdf]

Supplementary Information

Anosmin-1 activates vascular endothelial growth factor receptor and its related signaling pathway for olfactory bulb angiogenesis

Shoko Matsushima, Akio Shimizu, Manami Kondo, Hirotsugu Asano, Nobuhiro Ueno, Hironao Nakayama, Naoko Sato, Masahiro Komeno, Hisakazu Ogita and Misuzu Kurokawa-Seo

Supplementary Figure and Movie Legends

Supplementary Figure S1. Immunohistochemical analysis of OB in E10 chick embryo to check the specificity of the anti-anosmin-1 Ab. (a) Frozen sections of OBs were stained with the anti-anosmin-1 and anti-CD31 Abs in the presence of the recombinant chick anosmin-1 protein (quenching). **(b)** The OB samples were stained with the anti-CD31 Ab in the absence of the anti-anosmin-1 Ab. **(c)** The mouse OBs were stained with the anti-anosmin-1 Ab and anti-CD31 Abs. White dotted lines indicate the inner area of OB. Arrowheads indicate the CD31-positive staining. Scale bars: 200 μm.

Supplementary Figure S2. Tissue culture of chick OB by knockdown of anosmin-1. (a) Quantification of anosmin-1 mRNA expression levels by qPCR after transfection of control and anosmin-1 siRNA into OBs. **(b)** Immunofluorescence images of sprouting vasculature from OBs. The OBs isolated from the E10 chick embryo were cultured with the EBM-2 media for 3 days and were stained with phalloidin (green) and the anti-CD31 Ab (red). Dotted lines indicate the edge of OB. Yellow arrows in the bright field indicate the stretch of vessel-like structures. Scale bars: 100 μm. **(c)** Summary graphs for the lengths of sprouting vessel-like structures from OB. ** $P<0.01$ vs. siControl

Supplementary Figure S3. Endothelial cell migratory activity of anosmin-1 together with VEGF-A. (a) Transwell cell migration assay. HUVECs were seeded in the upper compartment, and were treated with the indicated concentrations of anosmin-1 and VEGF-A, or without reagents (0 nM). The cells that moved into the lower side of the chamber were counted and quantified. **(b)** Trypan Blue staining after treatment with PBS or 0.1% DMSO. Dead cells were stained in blue. **(c)** Summary graph of the percentage of live cells (Cell viability). **(d)** Phosphorylation of FGFR. Starved HUVECs were incubated with or without EGM-2 and/or SU5614 (10 μM) for 5 min. Cell lysates were analyzed by western blotting with the indicated Abs. **(e)** Knockdown of VEGFR2. HUVECs were incubated with 20 nM siRNA against VEGFR2 (siVEGFR2) or scramble RNA for 2 days. Cell lysates were analyzed by western blotting with the anti-VEGFR2 and anti-GAPDH Abs. **(f)** Transwell cell migration assay as described in **(a)**. VEGFR2-knockdown (siVEGFR2) or control (Scramble) HUVECs were used in this experiment. ** $P<0.01$ vs. without treatment (0 nM)

Supplementary Figure S4. Effects of anosmin-1 point mutations on cell migration. (a)

Expression of FGFR1 in PAE and PAE/KDR cells as well as HUVECs. Cell lysates were analyzed by western blotting with the anti-FGFR1 and anti-GAPDH Abs. **(b)** Generation of recombinant proteins of anosmin-1 harboring indicated point mutations. The purified recombinant proteins (0.5 μg each) were used for SDS-PAGE and visualized by SyproRuby staining. **(c)** Transwell cell migration assay. PAE/KDR cells were seeded in the upper compartment, and were treated with anosmin-1 WT, the indicated mutants or VEGF-A, or without reagents (0 nM). The cells that moved into the lower chamber were counted and quantified.

Supplementary Figure S5. BIAcore analysis for binding of anosmin-1 WT to VEGF-A. The data after the injection of anosmin-1 WT at the indicated concentrations are shown. Inset: Enlargement of the dotted rectangle.

Supplementary Figure S6 and S7. The entire images of all the western blots that were displayed in the manuscript. The dotted rectangles indicate the portions presented in each figure.

Supplementary Movie S1. The 3D image of OB vasculature. Frozen sections of OB at E10 were stained with the anti-anosmin-1 (green) and anti-CD31 (red) Abs. Pictures were captured in a Z-stack mode of confocal microscopy to generate the 3D image. One of the pictures was presented in Fig. 1B.

Supplementary Figure S1

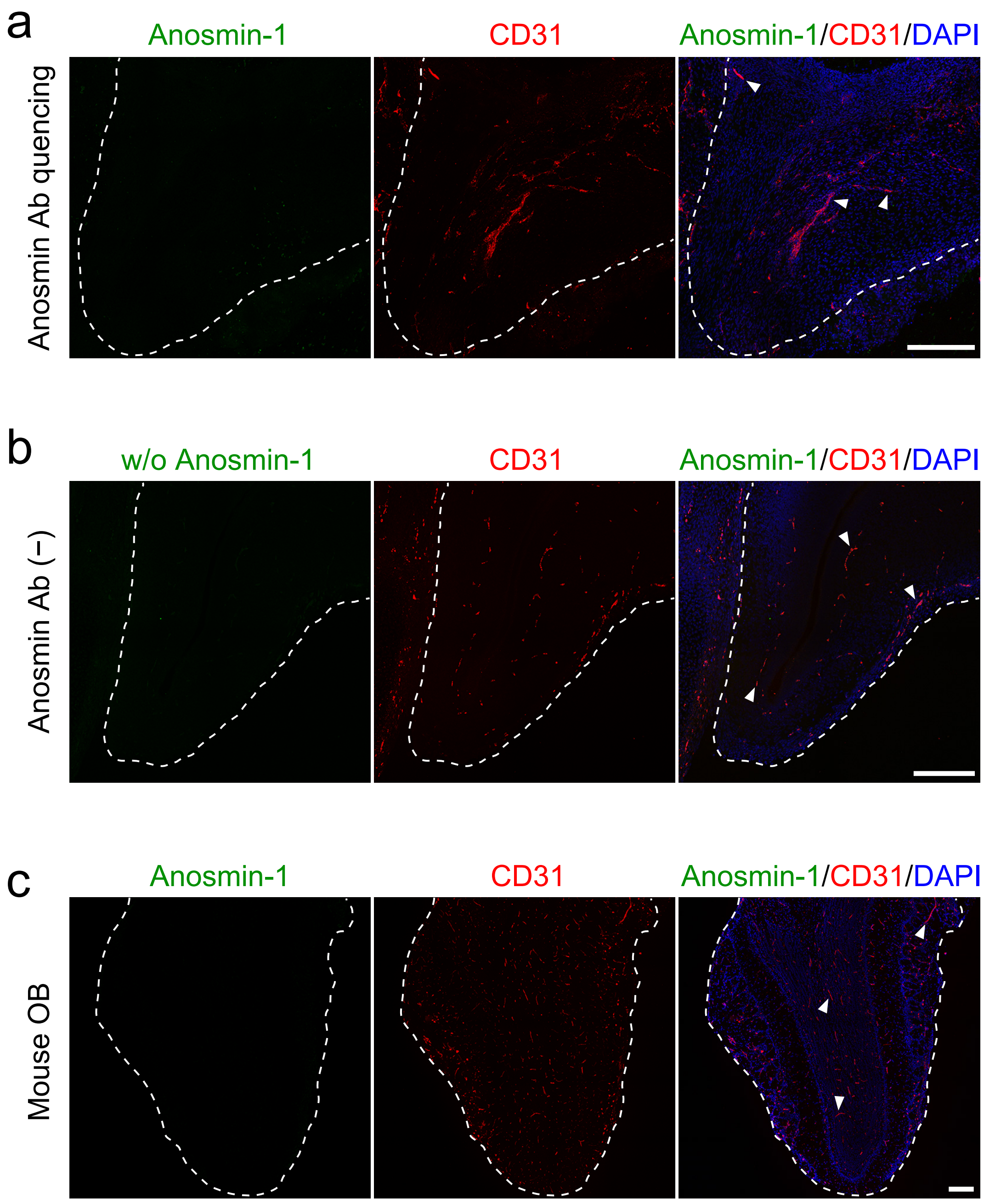

Supplementary Figure S2

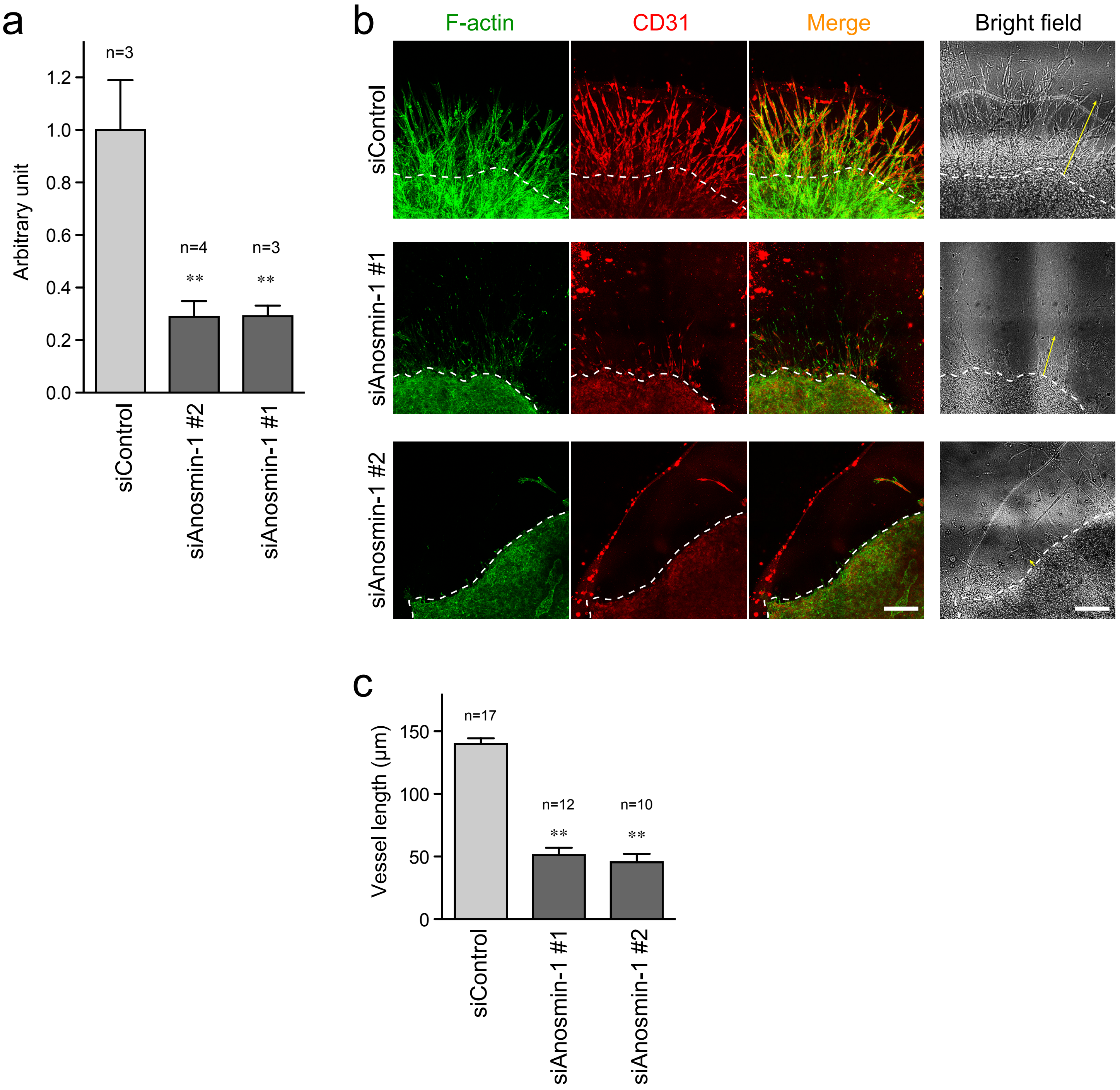

Supplementary Figure S3

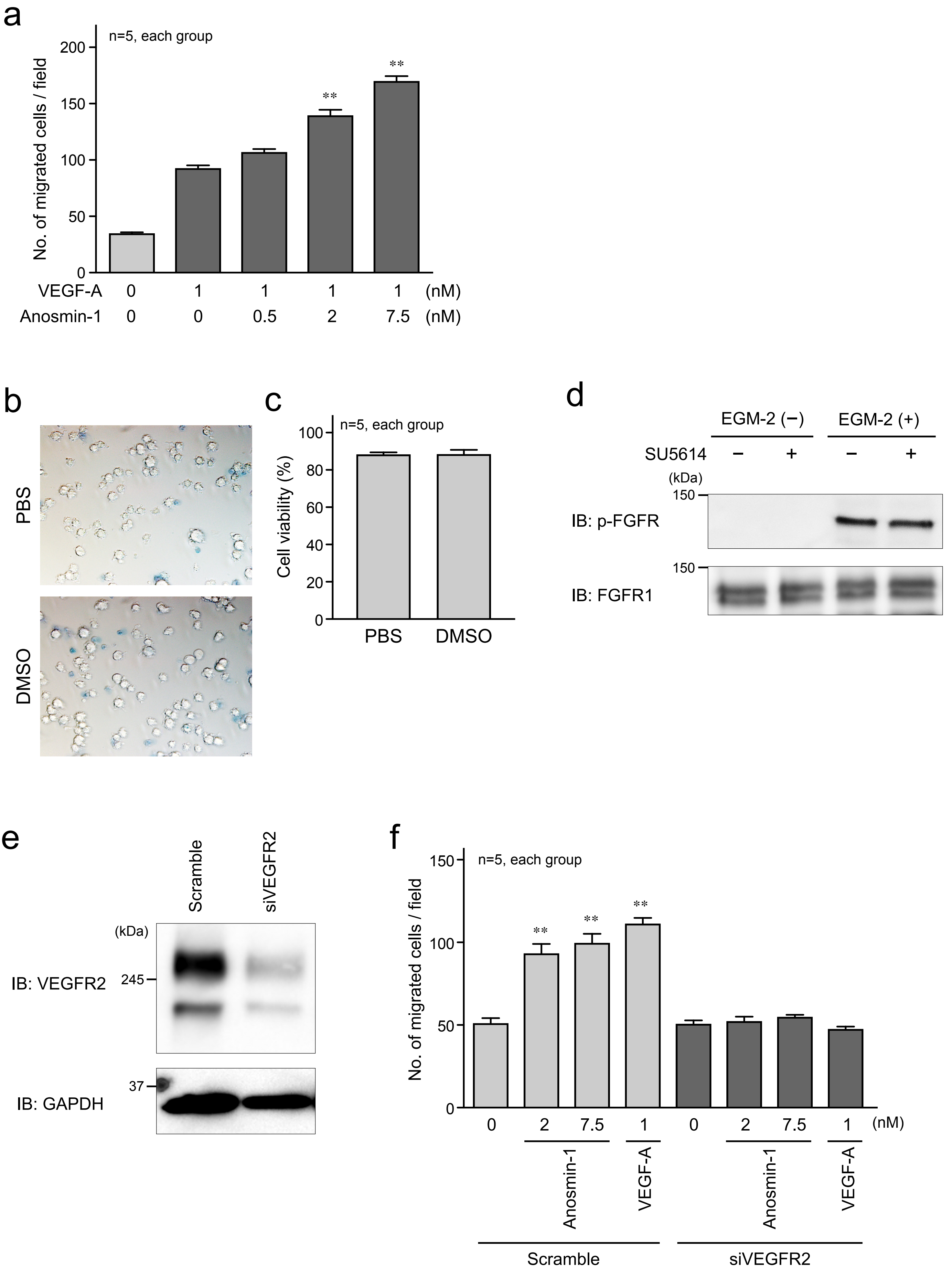

Supplementary Figure S4

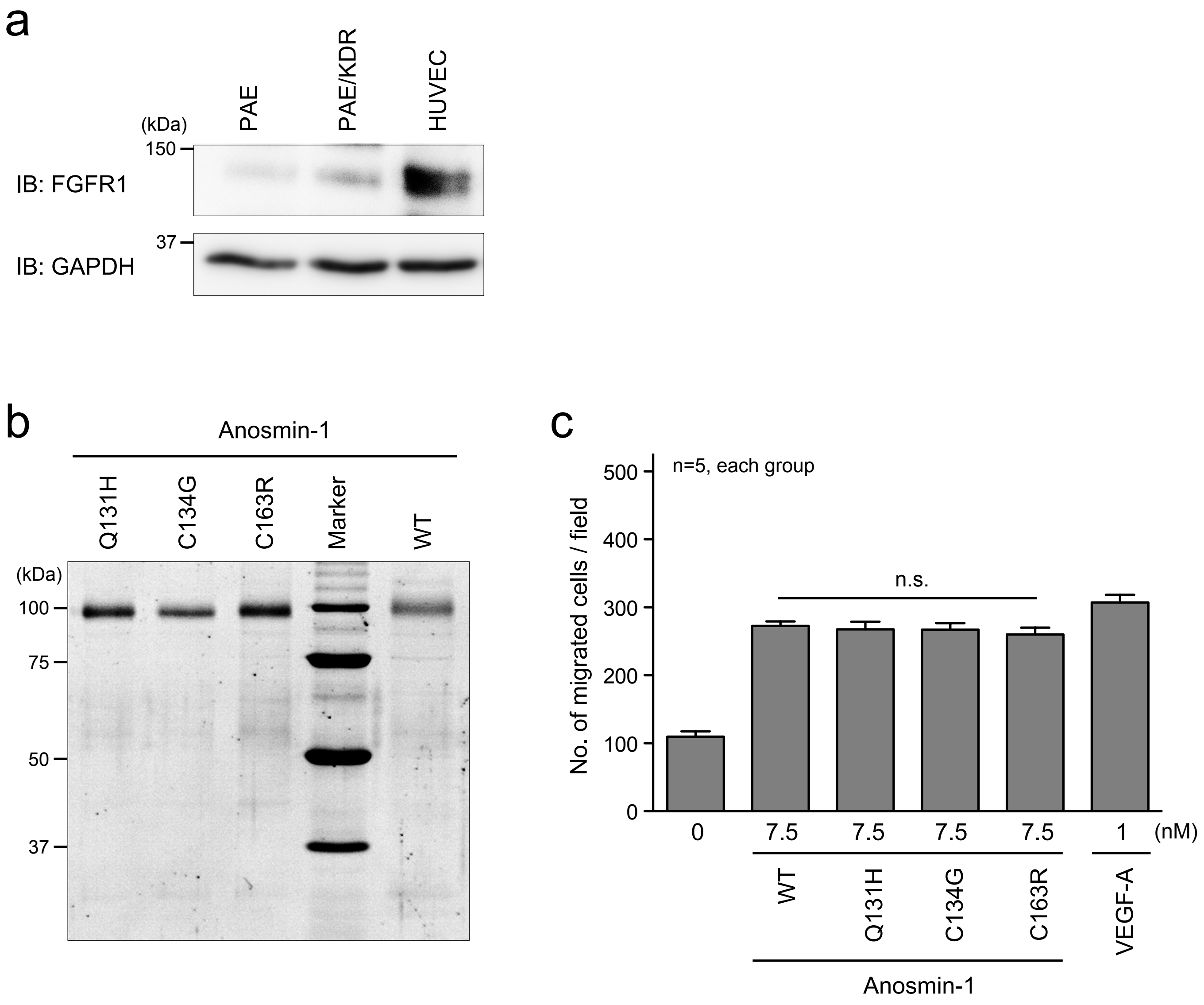

Supplementary Figure S5

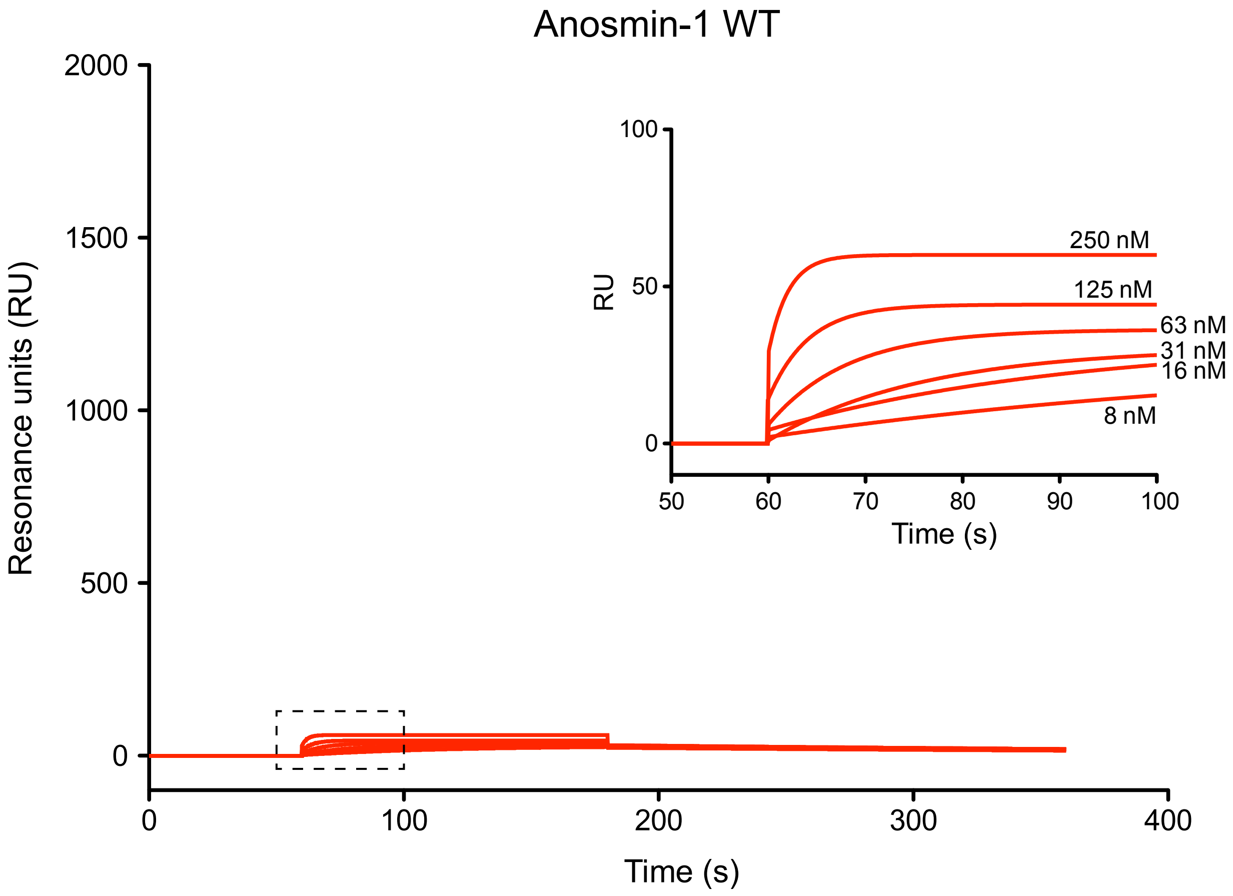

Supplementary Figure S6

Whole blots in Fig. 3a

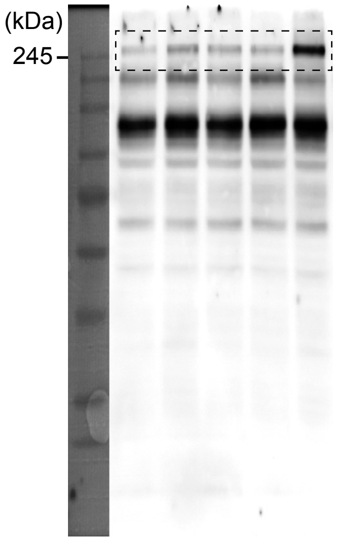

IB: p-VEGFR2
(p-Y1175)

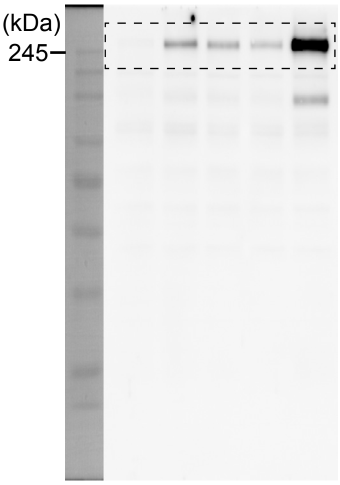

IB: p-VEGFR2
(p-Y1214)

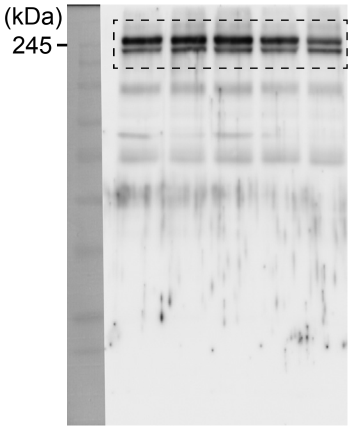

IB: VEGFR2

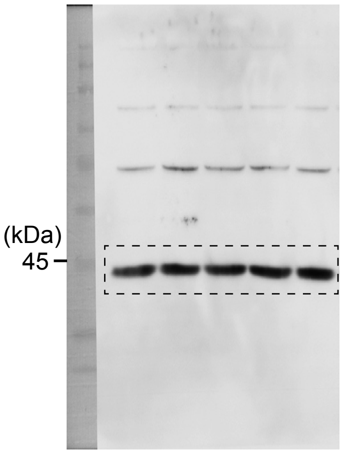

IB: β-Actin

Whole blots in Fig. 4a

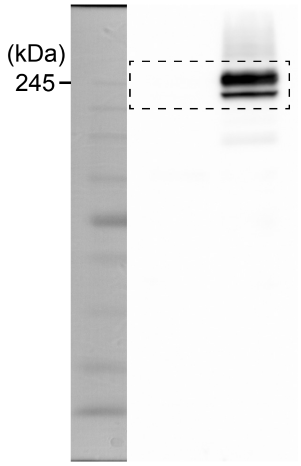

IB: VEGFR2

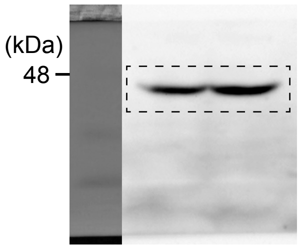

IB: β-Actin

Whole blots in Fig. 4f

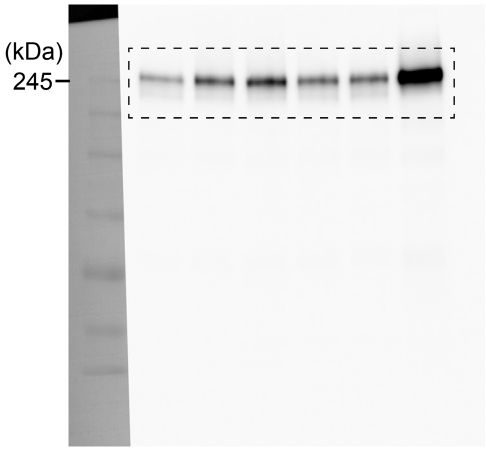

IB: p-VEGFR2
(p-Y1175)

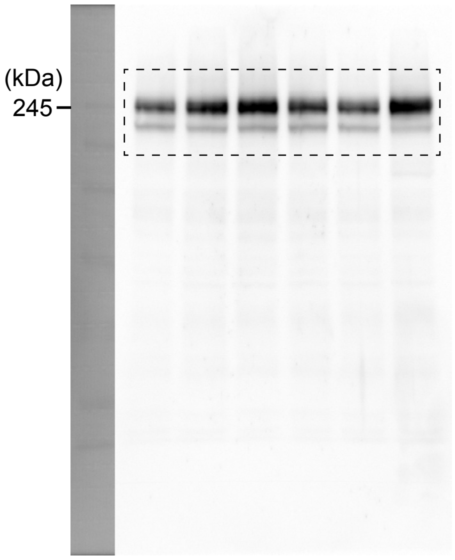

IB: p-VEGFR2
(p-Y1214)

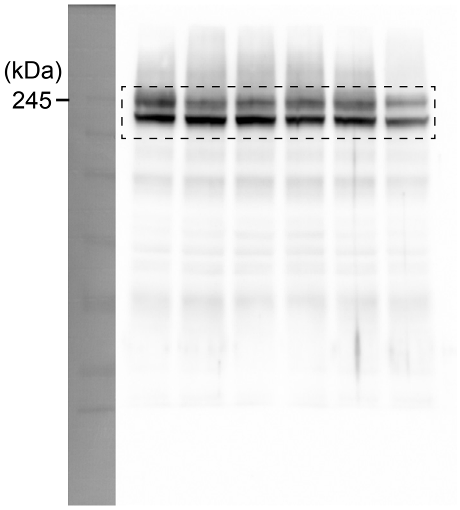

IB: VEGFR2

Supplementary Figure S7

Whole blots in Fig. 6a

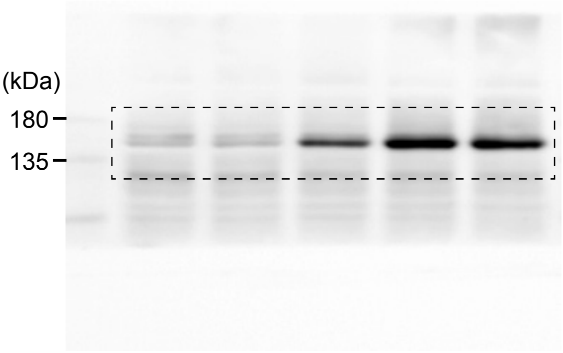

IB: p-PLCγ1

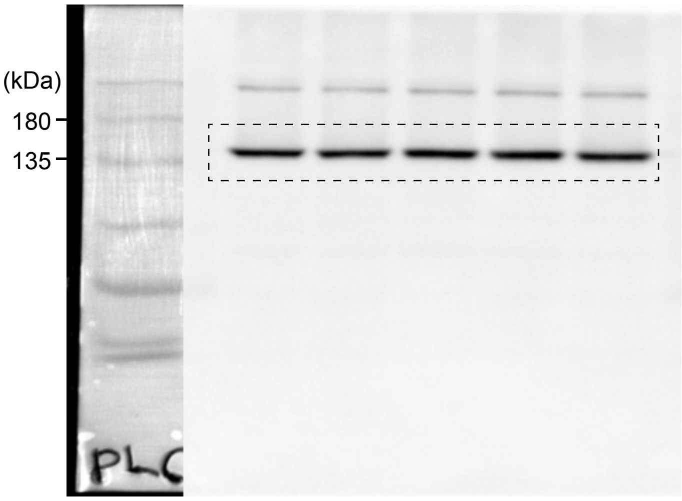

IB: PLCγ1

Whole blots in Fig. 6b

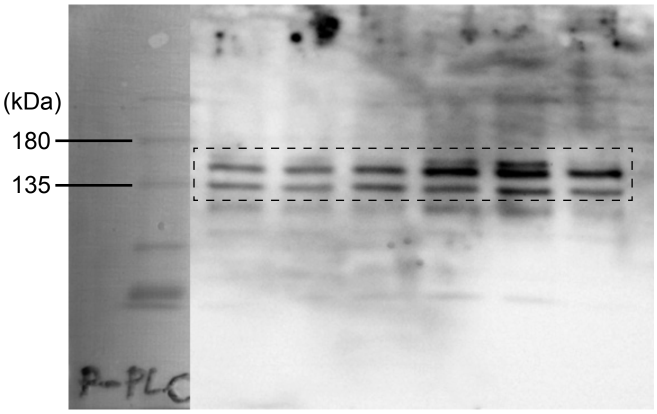

IB: p-PLCγ1

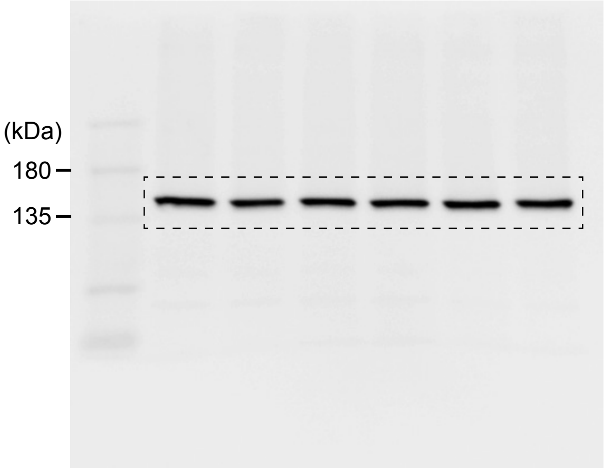

IB: PLCγ1

Whole blots in Fig. 6e

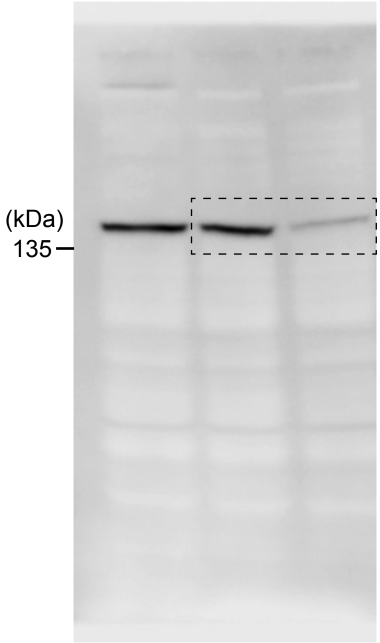

IB: PLCγ1

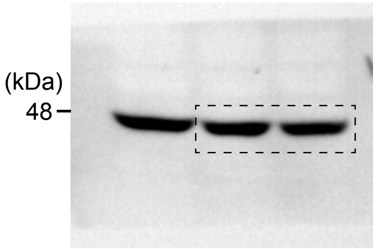

IB: β-Actin
